# Characterization and Applications of the Protein Corona Forming on the Naturally Occurring Nanoparticles, Low-Density Lipoproteins

**DOI:** 10.1021/acscentsci.6c00872

**Published:** 2026-09-14

**Authors:** Ding Ren, Lixia Wei, Simon Boutry, Melis Özkan, Ekaterina Poliukhina, Laia Simo Riudalbas, Krisztian Homicsko, Daniel E. Speiser, Jacques Fellay, Quy Khac Ong, Francesco Stellacci

**Affiliations:** † Institute of Materials, 27218 École Polytechnique Fédérale de Lausanne (EPFL), 1015 Lausanne, Switzerland; ‡ Global Health Institute, École Polytechnique Fédérale de Lausanne (EPFL), 1015 Lausanne, Switzerland; § Department of Oncology, 27213 University of Lausanne, 1015 Lausanne, Switzerland; ∥ Biomedical Data Science Center, Lausanne University Hospital and University of Lausanne, 1011 Lausanne, Switzerland; ⊥ Institute of Bioengineering, École Polytechnique Fédérale de Lausanne (EPFL), 1015 Lausanne, Switzerland

## Abstract

Low-density lipoproteins (LDL) are naturally occurring, monodisperse nanoparticles composed of proteins and lipids with an average diameter of 18–25 nm. Their biological functions such as cholesterol trafficking and their role in various diseases have been extensively studied. However, their properties as nanoparticles are seldom discussed. Here, we show that when isolated from biofluids, analogous to any synthetic nanoparticle, LDL carry a distinctive protein corona. The corona can be partly removed or preserved depending on the approach used to isolate them. Importantly, we find that proteomic analysis of this corona enables proof-of-concept separation between healthy individuals and patients with melanoma, colon carcinoma, or liver cancer. This approach provided clearer separation than direct proteomic analysis of serum because the corona of the LDL particle is relatively depleted of the serum-abundant proteins, most of which are only minor components of the corona. We postulate that proteomic analysis of lipoproteins can reveal disease-associated changes and that a deep understanding of the corona of these particles will advance the overall field of nanoparticles in medicine.

## Graphical abstract

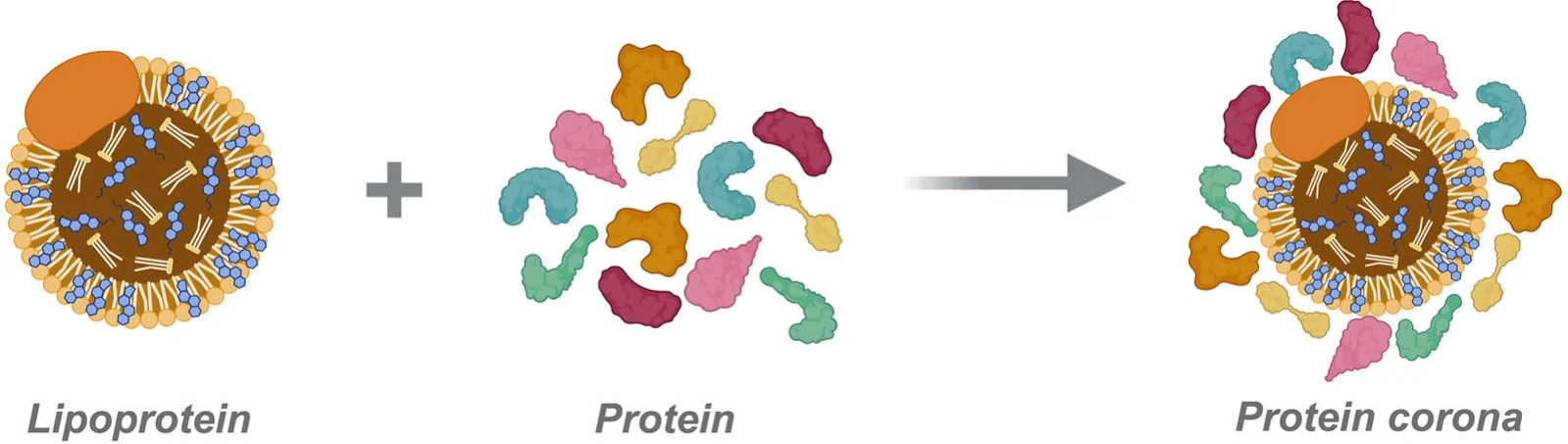

## Introduction

Upon contact with serum or other protein-rich biological fluids, the surface of synthetic nanomaterials (e.g., iron oxide,^1,2^ silica,^3,4^ gold^5^ or lipid^6,7^ nanoparticles) will be coated with a biologically active protein layer through nanoparticle–protein interaction. The layer was first named “protein corona” by Dawson and co-workers.^8,9^ The formation of a protein corona on nanoparticles poses complex challenges to the development of nanomaterials for medical purposes.^10^ While many studies focus on the decoration of nanomaterials with ligands to precisely modulate targeting and/or their interaction with specific biological entities,^11^ it is always necessary to consider the role of the protein corona to fully understand their complex interactions.^12^

The protein corona plays an important role (often undesirable) in a broad spectrum of biological effects, including cellular uptake, immune recognition, targeting efficiency, and biocompatibility.^13^ More recently, researchers have begun to recognize the potential of the protein corona,^2^ especially its use in personalized diagnostics and precision nanomedicine.^14,15^ The protein corona has also been exploited for disease biomarker discovery, where synthetic nanoparticles are used to enrich low-abundance proteins from biofluids and reveal disease-associated proteomic patterns.^16–18^ The identity and dynamics of the protein corona vary substantially,^19^ depending on nanoparticle properties such as size, shape, charge, and surface functionalization.^20,21^ Typically, two types of corona layers can be distinguished at the nanoparticle surface: a hard corona composed of tightly bound, slow-exchanging proteins that are difficult to separate, and a soft corona composed of loosely bound, rapid-exchanging proteins that can be separated more easily.^22^

Exosomes are naturally secreted nanosized vesicles highly involved in intercellular communication and have been shown to carry a native protein corona.^23,24^ The corona may be partly inherited from their parent cells but also continues to evolve, exchanging proteins with body fluids.^25^ However, the relatively low abundance of exosomes poses challenges for their isolation.^26^ Most reported isolation methods (including ultracentrifugation, precipitation, size-exclusion chromatography (SEC), or affinity capture) face poor reproducibility and technical variability, and often involve technically demanding procedures.^27,28^ Therefore, it is hard to standardize and quantitatively compare the protein corona on exosomes. Furthermore, due to the complexity of exosomes,^29^ it is unclear whether the detected proteins originate from the exosome corona, from the exosomes themselves, or from protein aggregates that are simply coisolated with exosomes.^30–33^ However, since exosomes were among the first naturally occurring biological nanoparticles reported to carry a protein corona,^24^ it opens the door for the idea that protein corona formation is not exclusive only to foreign nanoparticles in biological fluids but it may be a property of many-if-not-all naturally occurring nanoparticles.

Lipoproteins (LPs) are well-known naturally occurring nanoparticles for their biological functions in cholesterol trafficking.^34^ They consist of a heterogeneous family, including chylomicrons (CM; 75–1200 nm; unstable), very-low-density lipoproteins (VLDL; 30–80 nm), low-density lipoproteins (LDL; 18–25 nm), and high-density lipoproteins (HDL; 8–15 nm).^35^ LPs are produced in liver (VLDL and HDL) and intestine (CM and HDL), while LDL are generated from the conversion of VLDL that are unstable as such.^36^ LPs mainly consist of apolipoproteins, triglycerides, phospholipids and cholesterol.^37^ Since their discovery in the 1940s,^38^ LPs have been linked to a wide range of diseases. For example, elevated LDL levels are strongly associated with cardiovascular diseases^39^ (e.g., atherosclerosis and coronary artery disease) and elevated VLDL levels are associated with metabolic syndrome and type 2 diabetes.^40^ Lipoproteins are also linked to cancer, as studies have shown that they transport lipids to tumor cells to support their growth and proliferation.^41,42^ However, compared to their widely studied biological functions, LPs’ nanoscale properties are rarely discussed, though Munter and co-workers raised the possibility of LPs forming corona with surrounding proteins.^43^

In this work, we focus on LPs’ interaction with surrounding proteins in the bloodstream and show that LDL have a protein corona when extracted from serum or plasma. To our knowledge, this is the first direct characterization of a protein corona on isolated LDL from human biofluid. We selected LDL to study corona formation because of the low stability of VLDL and CM, as well as the small size and low extraction yield of HDL. We find that precipitation induced by poly­(ethylene glycol) (PEG) 10K^44^ followed by fast-protein liquid chromatography (FPLC) purification preserves the exchangeable proteins associated with LDL, while density-gradient ultracentrifugation (DGUC) removes much of the exchangeable proteins and results in LDL with only a hard corona. These two LDL states show differences in hydrodynamic size, density and protein composition. LDL carrying the exchangeable corona are larger and contain more proteins while the protein composition of LDL without soft corona is dominated by apolipoprotein B-100 (ApoB-100). Hereafter, we refer to LDL without soft corona simply as LDL and to LDL with soft corona as ProLips (protein-rich lipoprotein nanoparticles). In the tested clinical human samples, in ProLips, the soft corona was poor in the abundant blood proteins such as albumin that often hinder the detection of rare proteins. Therefore, the analysis of ProLips provides broader insight into the broad spectrum of proteins of a given individual. We found that ProLips extracted from serum or plasma enabled proof-of-concept separation between healthy donors and colon carcinoma patients through the detection of 166 up- or down-regulated proteins among 477 proteins. The same approach also worked for melanoma and liver cancer, where for either cancer type, more than 200 of the 600+ detected proteins were significantly differentially expressed. Subsequent analysis on 31 melanoma and 19 healthy samples allowed us to identify a set of six proteins as candidate melanoma-associated biomarkers. This new biomarker set was then evaluated in an independent set of nine melanoma samples in which it separated the melanoma and healthy-control groups without misclassification.

## Results

### Extraction and Characterization of LDL

To obtain LDL depleted of as much exchangeable corona as possible, LPs were extracted via a commonly used DGUC approach^37,45^ (as described in detail in the Supporting Information) with modifications. Different concentrations of potassium bromide (KBr) solutions were prepared to create a density gradient. Healthy pooled serum was layered at the bottom with an adjusted relative density at 1.21, while three different density gradients were layered above (1.006, 1.019 and 1.063, corresponding to density of VLDL, LDL and HDL, respectively; Figure 1a). After high-speed ultracentrifugation which generated enough force to strip off most of the proteins potentially associated with LPs, LP bands were separately collected and further purified via size-exclusion chromatography (SEC) coupled with fast-protein liquid chromatography (FPLC). Figure 1b shows the corresponding fractograms, where LDL and HDL showed sharp peaks at 8–9 and 9–10 mL, respectively. VLDL, because of their low concentration and low protein content, were not clearly detected in the fractograms. DLS curves (Figure 1c) and TEM images (Figure 1d–f) showed the number-wise average hydrodynamic diameters of VLDL, LDL and HDL at 32 ± 10, 18 ± 5, and 9 ± 2 nm, respectively, in good agreement with previous reports.^43,46^ The polydispersity of VLDL (0.52) is much larger than those of LDL (0.27) and HDL (0.23). Sodium dodecyl sulfate–polyacrylamide gel electrophoresis (SDS-PAGE) further supports the extraction of LDL and HDL, as the observed band patterns include bands at apparent molecular weights consistent with ApoB and ApoA1, respectively (Figures 1g and S1).

**Figure 1:**
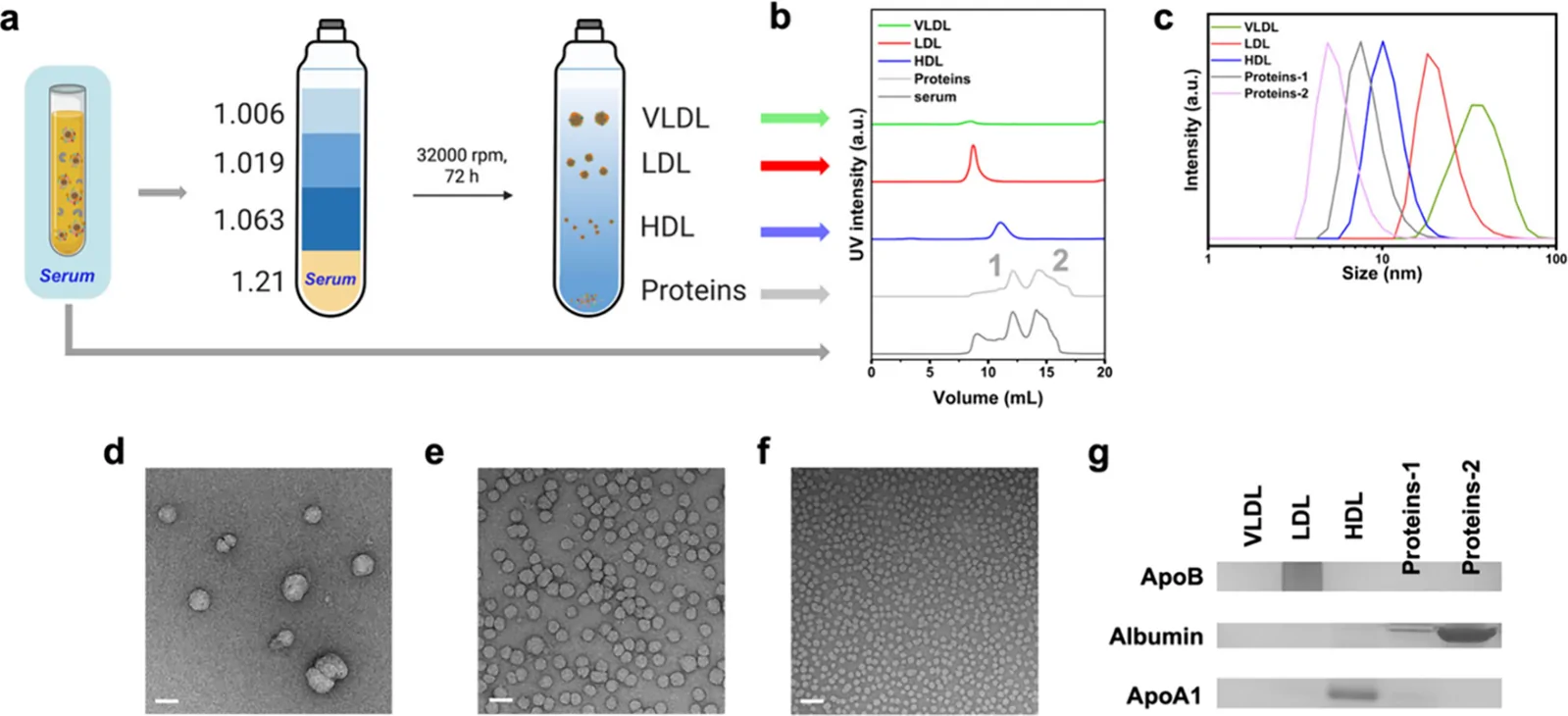
Purification and characterization of LPs. (a) Schematic illustration of DGUC purification of serum. (b) FPLC fractogram of LPs. The peaks corresponding to different protein-containing bands are labeled. (c) DLS curves of LPs. (d–f) TEM images of VLDL, LDL, and HDL, respectively. Scale bar: 50 nm. (g) SDS-PAGE gel showing bands at apparent molecular weights consistent with ApoA1, ApoB, and albumin in VLDL, LDL, HDL, and proteins after DGUC.

### Extraction and Characterization of ProLips

ProLips were extracted via a much milder approach based on the addition of poly­(ethylene glycol) with a molecular weight 10,000 (PEG 10K) to the serum (Figure 2a). PEG 10K was chosen to induce lipoprotein precipitation via depletion forces,^47,48^ reasoning that this approach would precipitate ProLips colloidally without creating or disrupting the associated proteins. After diluting serum 10 times with phosphate-buffered saline (PBS) and filtering away large aggregates, we added a 50% m/v PEG 10K solution with a volume equal to 1/5 of the treated serum. The mixture was incubated and centrifuged to separate the precipitate from the supernatant. The former was then redispersed in PBS and the procedure was repeated. Similar to LPs, the newly obtained suspension was then purified via FPLC-coupled SEC. Other polymers [poly­(vinyl alcohol), polyacrylamide, and poly­(*N*-isopropylacrylamide)] were also used to help precipitation, but we obtained ProLips only in low yields if at all (Table S1 and Figure S2). The protein concentration of dissolved precipitates is approximately 2.5 mg mL^–1^, whereas the protein concentration of serum is approximately 50 mg mL^–1^.

**Figure 2:**
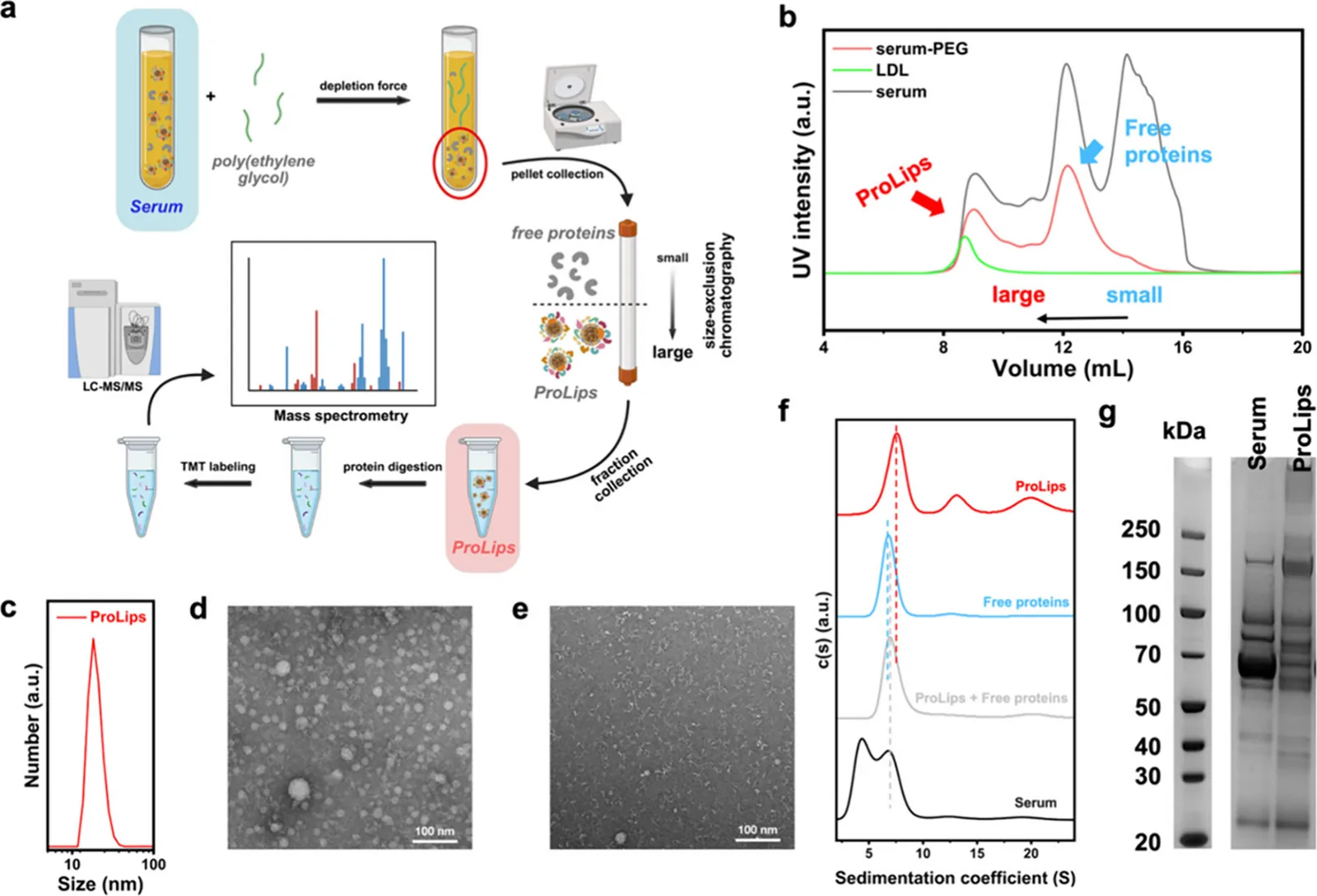
Purification and characterization of ProLips. (a) Schematic workflow of the ProLips approach and mass spectrometry for ProLips proteome analysis. Human serum was first coincubated with PEG to precipitate ProLips and free proteins. Following isolation of the pellet from the supernatant by centrifugation, the pellet was redispersed in PBS and subjected to SEC to separate free proteins from ProLips. Proteomic analysis started with the digestion of proteins from ProLips. The resulting peptides were then labeled with tandem mass tags and subjected to LC–MS/MS analysis using DDA top-speed mode. (b) FPLC fractograms of PEG-precipitated serum, serum, and LDL. (c) DLS curve of ProLips. (d and e) TEM images of ProLips and free proteins, respectively. (f) AUC curves of ProLips, free proteins, PEG-precipitated serum, and serum. (g) SDS-PAGE image of serum and ProLips.

Figure 2b illustrates a typical FPLC fractogram of the sediment after PEG precipitation (red). Two main fractions are observed at approximately 9 and 12–13 mL and can be collected separately. The FPLC fractogram of serum was shown in dark gray, which comprises the same two major peaks plus a third peak (14–15 mL) that is dominated by albumin (Figure S3a,c,f). Notably, besides the third peak, the serum and precipitate fractograms are essentially identical, indicating that PEG precipitation preserves the LDL-associated proteins, and most albumin remains in the supernatant. We assign the first fraction in the fractograms to ProLips because when DGUC-purified LDL is analyzed by FPLC on the same column, it yields a single peak at 8–9 mL (Figure 2b, green). The protein concentration of ProLips solution is approximately 1.1 mg mL^–1^, corresponding to an overall protein recovery of approximately 2%. DLS measurements on the first FPLC fraction (Figure 2c) revealed a major component with a hydrodynamic diameter of 20 ± 5 nm, which is also confirmed by negative staining TEM images (Figure 2d). This is comparable to LDL (18 ± 5 nm), yet slightly higher, consistent with the presence of a protein corona. The second fraction is assigned to free proteins, as corroborated by negative staining TEM images (Figure 2e) that lack nanoparticles. A further confirmation that the first fraction is composed of ProLips comes from analytical ultracentrifugation in sedimentation velocity mode (AUC-SV) (Figure 2f), showing a sedimentation coefficient (SC) of ProLips of approximately 7 S, in line with the reported value of LDL.^49^ A more complete analysis of the AUC-SV curves of serum, ProLips, and free proteins revealed that ProLips are present in serum before the addition of PEG. In fact, the serum AUC trace (black in Figure 2f) can be construed as the sum of the ProLips and the free proteins traces (red and light blue in Figure 2f, respectively).

### Analysis of ProLips Composition

To elucidate the components of ProLips (i.e., the first fraction in FPLC purification of the PEG precipitate), we subjected them to the same DGUC procedure used to extract LPs from serum. LP bands were collected at the same buoyant-density positions in the tube as in serum DGUC and labeled accordingly (e.g., P-VLDL for the VLDL band, P-LDL for the LDL band). Each band was further purified and analyzed by FPLC to compare LP abundance. By comparing FPLC fractograms of the serum-extracted (Figure 1a,b) and ProLips-extracted (Figure 3a,b) LPs, we observe that the LDL band is the most abundant in both cases. The main difference lies in the HDL band, which is substantial in the former case and absent in the latter. Consistently, in ProLips-DGUC fractions, all LP bands other than LDL were present at only trace levels, while the protein bands remained substantial. DLS showed that the hydrodynamic diameter of LDL and P-LDL are both 18 ± 5 nm (Figure 3c). The close agreement indicates that DGUC isolates LDL particles of essentially the same hydrodynamic size (Figure 3d), regardless of the upstream purification. AUC-SV on ProLips (Figure 2f) showed a major peak, indicating a single particle population, albeit within the expected polydispersity, and two side peaks that can be attributed to aggregation into dimers and trimers. DGUC thus separates ProLips mainly in two main components–LDL and proteins–suggesting that DGUC strips the associated proteins from LDL. SDS-PAGE performed in a lower concentration further supports the interpretation: the gel image of P-Proteins (Figure 3e) closely matches that of ProLips, except for a high-molecular-weight band consistent with ApoB (marked with a yellow arrow) which was observed only in ProLips and P-LDL.

**Figure 3:**
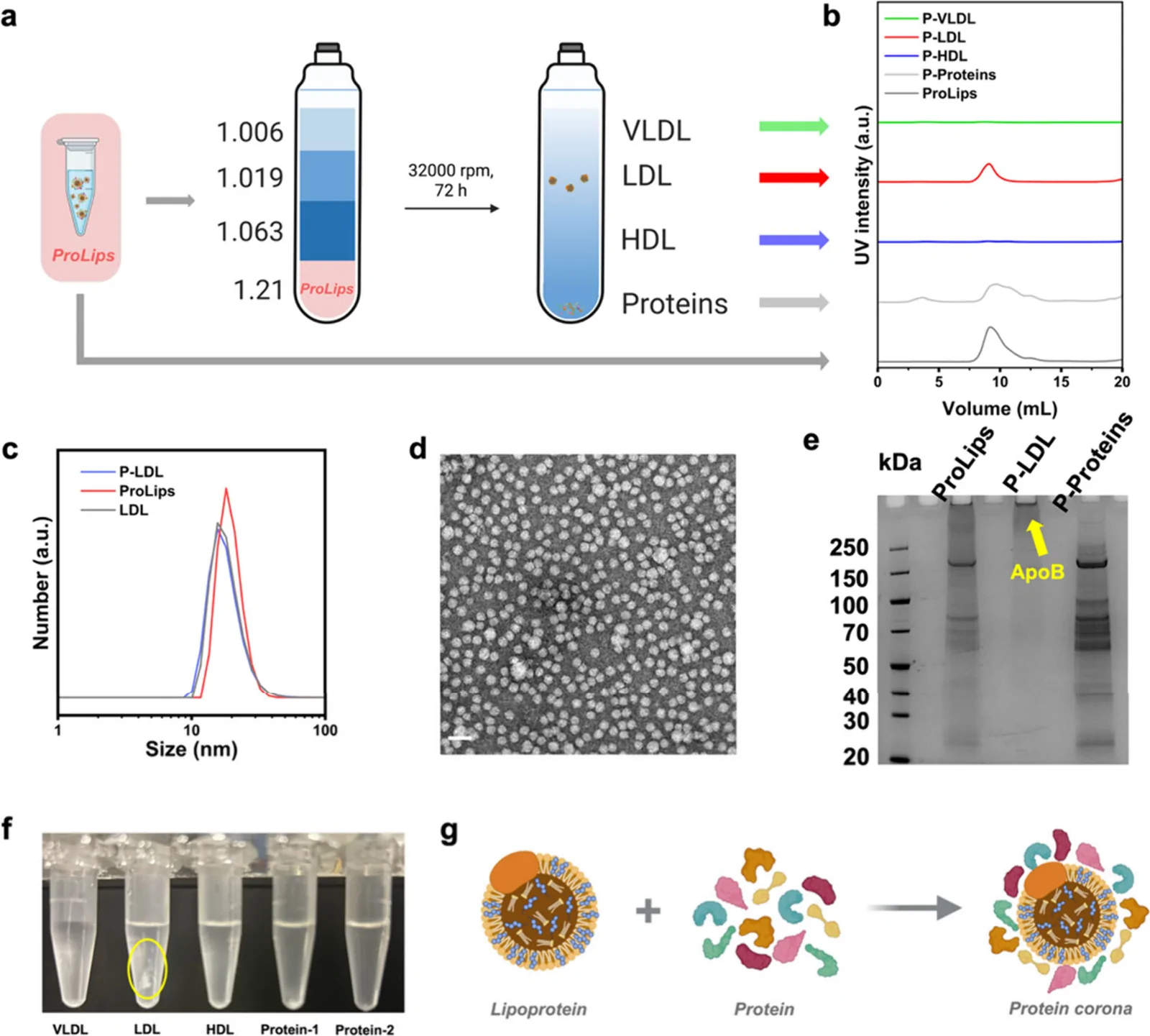
Corona study on ProLips. (a) Scheme of DGUC purification on ProLips. (b) FPLC fractogram of P-LPs. (c) DLS curves of P-LDL, ProLips, and LDL. (d) TEM image of P-LDL. Scale bar: 50 nm. (e) SDS-PAGE image of P-LDL, ProLips, and P-Proteins. (f) Photograph of VLDL, LDL, HDL, and two protein fractions (Protein-1 and Protein-2) after incubation with PEG and subsequent centrifugation under the same conditions. A visible precipitate is observed only for LDL (yellow circle). (g) Schematic illustration of protein corona formation around lipoproteins.

To test whether PEG precipitation results from nonspecific depletion forces on nanoparticles, or from possible PEG–protein interactions, we applied the same PEG-precipitation protocol to (i) LDL and (ii) serum-extracted proteins adjusted to the same protein concentration. After incubation and centrifugation, a visible precipitate formed in the PEG–LDL mixture, whereas no precipitation was observed in the PEG–protein mixture (Figure 3f). This suggests that PEG does not precipitate proteins alone under our conditions; rather, precipitation requires the presence of nanoparticles (LDL) and is consistent with the contribution of LDL–protein interactions. (Figure 3g)

We next compared the detailed protein composition of serum, LDL, and ProLips by label-free proteomics, using spectral counts as a proxy for relative protein abundance (Table S2 for the top abundant proteins). In serum, albumin ranked first and accounted for >20% of spectra, whereas its spectral percentage decreased to <5% in ProLips and LDL, indicating efficient exclusion of albumin in ProLips extraction. In LDL, ApoB represented the dominant component (approximately 50%), in agreement with the SDS-PAGE gel image (Figure 1g), followed by ApoA1 and C3. In contrast, the protein composition was more heterogeneous in ProLips, with A2 M contributing the highest spectral counts, followed by ApoB and C3. We can further substantiate our conclusions on the protein compositions of ProLips and whole serum when comparing their gel images (Figure 2g). The albumin band is dominant for serum, while we see a more even distribution of proteins for ProLips.

Taken together, these results support that ProLips are LDL particles coated with a protein corona. We find no evidence of major purification-induced corona formation and the similarity between AUC profiles of LDL and ProLips strongly suggests that ProLips are already present in serum. DGUC, in turn, removed much of the exchangeable associated proteins from LDL. Importantly, this protein corona is not rich in the most-abundant serum proteins although we do detect albumin. We therefore speculate that the physicochemical properties of LDL may limit association with highly abundant serum proteins and that the ProLips proteome may encode information about donors’ health status. This could result from specific binding interactions, or more generally from ProLips acting as nanoscale “sponges” that sample the circulating proteome while filtering out at least albumin.

### Proteomics

The corona of synthetic nanoparticles has been shown to contain cancer-associated proteomic information.^16–18^ Based on these observations, we hypothesize that the LDL-associated protein corona may also reflect changes related to cancer. This possibility is further supported by the broad biodistribution and high concentration of LDL in blood, as well as the reduced representation of highly abundant blood proteins (e.g., albumin) in the LDL-associated protein fraction. In our first proof-of-concept cancer-related proteomics run, we compared the ability of ProLips and serum proteomics to distinguish between healthy controls and melanoma patients. Five serum samples were obtained from five late-stage melanoma patients and compared to five samples obtained from one pooled serum of healthy donors. Proteins were enzymatically digested into peptides, labeled with TMTpro 10plex reagents, fractionated into 12 fractions under an off-gel system, and analyzed by liquid chromatography-tandem mass spectrometry (LC–MS/MS) using a data-dependent acquisition (DDA) top-speed mode. Across all whole-serum samples, 636 proteins were identified. Two-dimensional principal component analysis (2D-PCA) of whole-serum samples did not clearly separate melanoma from healthy individuals (Figure 4a). As shown in the volcano plot presented in Figure 4b, among the 636 detected proteins, only 21 were significantly up-regulated (FDR < 0.05) and 19 were down-regulated in melanoma serum compared to healthy serum. In stark contrast, ProLips analysis revealed much clearer distinctions while maintaining the broad detection of proteins (615 proteins). 2D-PCA clearly segregated healthy and melanoma ProLips into distinct groups (Figure 4c). In Figure 4d, the volcano plot showed that in the case of ProLips there were 361 differentially expressed proteins (DEPs). Among them, 200 proteins were significantly up-regulated and 161 proteins were down-regulated. Protein intensity correlation analysis further demonstrated the difference in protein composition between ProLips and serum. The protein profiles of melanoma and healthy serum were highly correlated (Pearson’s *r* = 0.991), so was the correlation for ProLips profiles (Pearson’s *r* = 0.966; Figure S4a,b). The lower correlation observed for ProLips may partly reflect the reduced contribution of highly abundant serum proteins^50^ (Figures S4c,d and S5).

**Figure 4:**
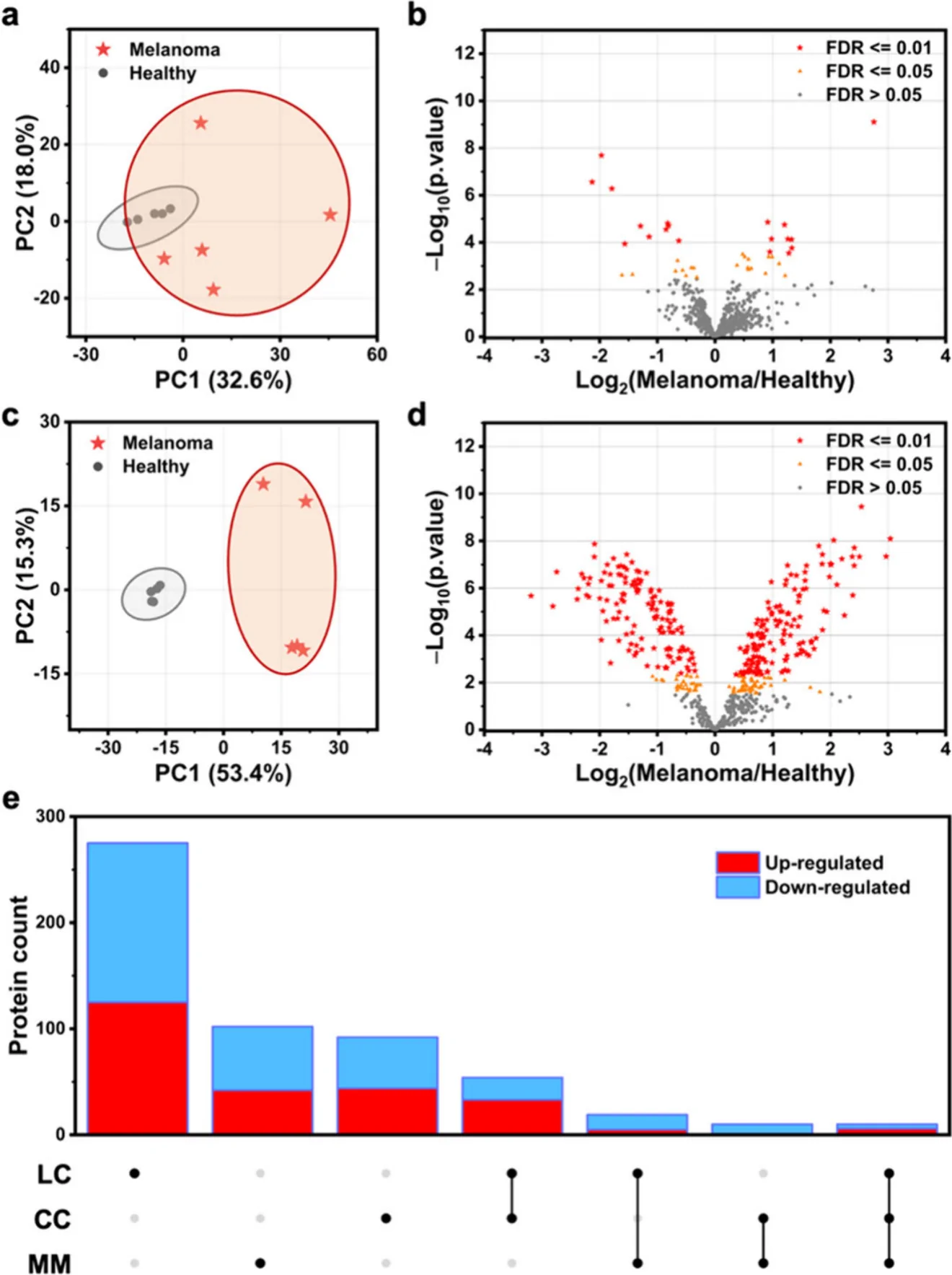
Proteome analysis on cancer vs healthy. (a) PCA on melanoma serum vs healthy serum. (b) Corresponding volcano plot of protein expression comparison. Each dot represents a protein here. (c) PCA on melanoma ProLips vs healthy ProLips analysis. (d) Corresponding volcano plot of protein expression comparison. (e) UpSet plot showing the overlap of significantly up- or down-regulated proteins liver cancer (LC), colon carcinoma (CC), and melanoma (MM).

Comparisons using individual samples were also performed between patients with melanoma, colon carcinoma, or liver cancer and healthy individuals. Five melanoma patients were compared to five healthy individuals. ProLips were extracted and analyzed in the same way as the first study. PCA clearly showed the separation of the two cohorts. Among the 700 proteins detected, 141 proteins were significantly up- or down-regulated. The smaller number of differentially expressed proteins (DEPs) may reflect the interindividual variability captured by using the individual healthy samples. In the colon-carcinoma-versus-healthy comparison, seven patients as a group were compared to eight healthy individuals. Among the 477 proteins detected, 166 proteins were significantly up- or down-regulated. In the liver-cancer-versus-healthy comparison, seven patients as a group, were compared to ten healthy individuals. Among the 628 proteins detected, 169 were up-regulated and 189 were down-regulated. The high proportion of DEPs may be related to the central role of the liver in lipoprotein metabolism, as liver cancer has been associated with altered profiles of lipids, lipoproteins and apolipoproteins.^51^ Figure 4e shows the overlap of DEPs among the three cancer types. Only a few proteins are significantly up-regulated or down-regulated in all three cancer types, suggesting that most DEPs are not pan-cancer but specific to a certain cancer type.

### Biomarker Discovery

We expanded our analysis in an attempt to identify a group of proteins as potential biomarkers for melanoma. A total of 31 melanoma samples and 19 healthy samples were processed with the same ProLips extraction protocol and analyzed through multiple TMT runs (Table S4 for sample information and Figure S8). DEPs were identified for each run, and proteins were grouped based on their occurrence across these sets. Specifically, we examined which proteins were consistently identified as DEPs in one or more of the five independent runs. Using thresholds of 1, 2, 3, 4, and 5 occurrences, the resulting protein groups (labeled DEP-1, DEP-2, ..., DEP-5) contained 95, 22, 14, 7, and 1 proteins, respectively. These protein groups were applied to cluster the samples in those runs based on normalized expression and the average clustering accuracy was calculated. Neither DEP-1 nor DEP-5 achieved satisfactory clustering performance, with average accuracies below 80% (Figure 5a). The poorer performance of DEP-1 likely reflects the presence of proteins identified as differentially expressed in only one single proteomic run, which are expected to represent less reproducible or run-specific signals. Conversely, DEP-5 was too restrictive, retaining only one consistently detected protein and therefore failing to capture the broader biological variation associated with melanoma. In contrast, DEP-2, DEP-3, and DEP-4 consist of proteins repeatedly identified across independent runs, enriching for more robust biomarkers while excluding unstable features. This suggests that biomarker robustness depends on the number of proteins included as well as their reproducibility across independent proteomic runs.

**Figure 5:**
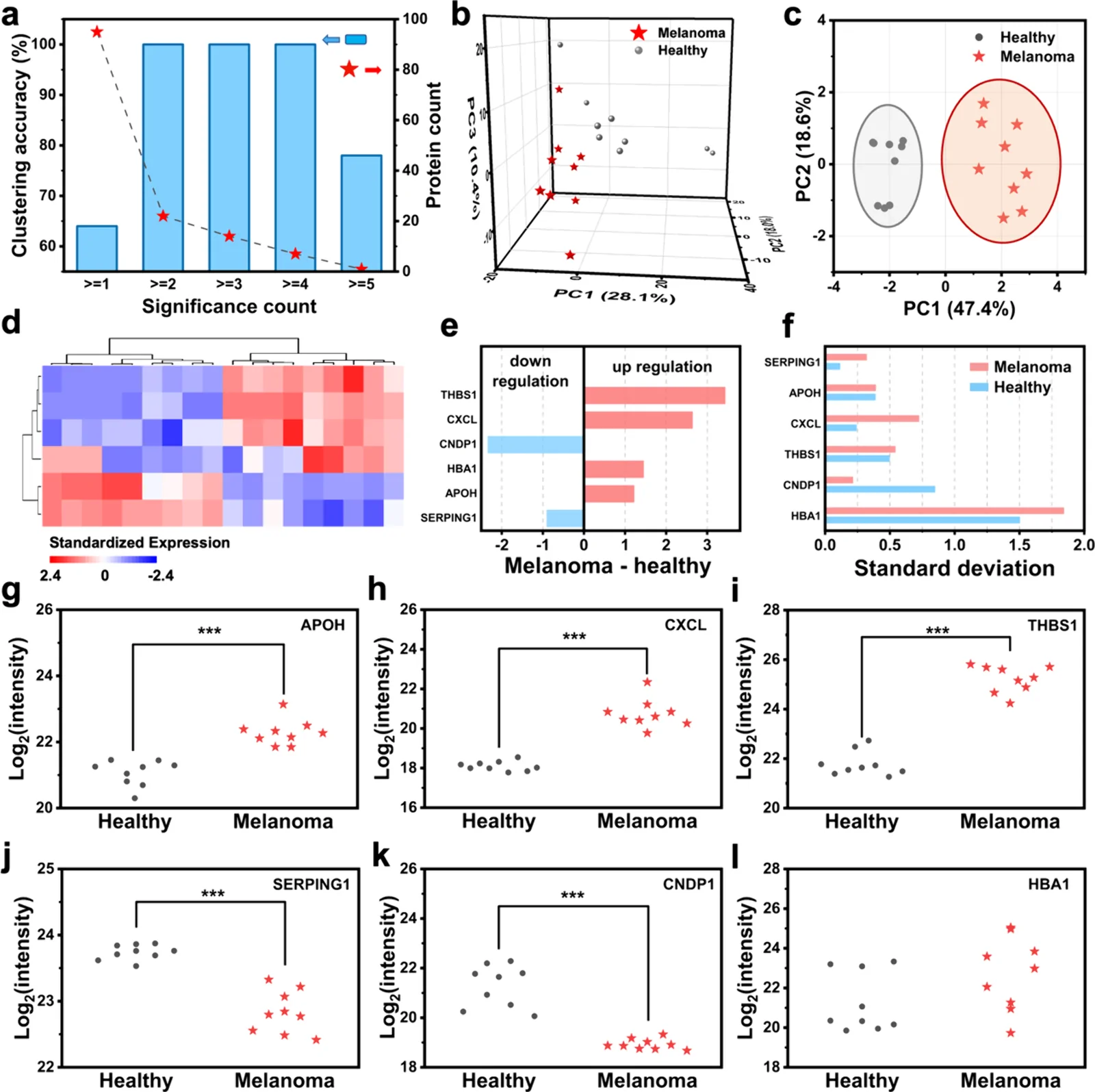
Melanoma biomarker discovery and validation. (a) Sizes of different protein groups and their corresponding average clustering accuracies. The horizontal axis is the threshold of protein existence in different DEP lists. (b) 3D-PCA of the validation group based on all detected proteins. (c) 2D-PCA of the validation group based on proteins from DEP-4. The gray and orange ovals are visual guides. (d) Heatmap of protein standardized to a normal distribution. Each row represents one protein, and each column represents one sample from the validation run. (e) Mean difference of each protein from DEP-4 in the validation run. (f) Intragroup standard deviation of each protein from DEP-4 in the validation run. (g–l) Expression of each protein from DEP-4 in each sample from the validation run. Pairwise correlations are shown where asterisks represent *p*-value thresholds: *p < 0.05, **p < 0.01, and ***p < 0.001.

In order to validate these groups, a separate TMT-labeled LC–MS/MS analysis was performed on ProLips. Nine samples were derived from nine melanoma patients. As a healthy control group, we took nine samples from four pooled serum samples (see the Methods section). PCA separated the groups only when a three-dimensional analysis was performed (Figures 5b, S9, and S10), potentially because the analysis also captured differences among the pooled healthy samples. If PCA is performed on the melanoma patients against samples from a single pool then a two-dimensional analysis suffices (Figure S11). Nevertheless, a 2D-PCA on all 18 samples based solely on DEP-4 (seven proteins) distinctly separates melanoma patients from healthy samples (Figure 5c) with PC1 as the clear separator. In hierarchical clustering, once again, DEP-2, DEP-3, and DEP-4 showed no misclassification, while clustering based on all detected proteins misclassified 7 out of 18 samples (Figures 5d and S12). Silhouette analysis was applied to assess clustering quality. In the validation set, the average silhouette values of DEP-2, DEP-3, and DEP-4 were 0.48, 0.50, and 0.56, respectively, indicating that DEP-4 offers the most robust separation of melanoma and healthy cohorts. Clustering based on the protein subgroup obtained by subtracting DEP-4 from DEP-3 revealed one misclassified melanoma sample, also suggesting that DEP-4 is a more representative melanoma biomarker set than DEP-3. It should be noted that DEP-4 correctly clustered the samples despite the fact that one of the seven proteins that make up the DEP-4 set was not detected in this experiment. Collectively, these results support the six remaining proteins in DEP-4 as a candidate protein group for melanoma detection.

To evaluate the contribution of each protein in clustering, we calculated the mean difference and standard deviation of each protein from DEP-4. Among the six proteins detected, THBS1, CXCL, HBA1, and APOH are up-regulated in ProLips from the melanoma cohort (proteins are listed in decreasing order of mean difference), while CNDP1 and SERPING1 are down-regulated (Figure 5e). Since a higher mean difference indicates greater contribution in clustering, THBS1 emerged as a key protein in group separation. Additionally, SERPING1 demonstrates the lowest intracohort standard deviation, indicating the most consistent expression levels within groups, while HBA1 showed the highest variation (Figure 5f). The distribution of log_2_(normalized intensity) values for the six proteins further supported these findings for THBS1, CXCL, and APOH that were significantly up-regulated (FDR < 0.001) and for CNDP1 and SERPING1 that were significantly down-regulated in the validation run (Figure 5g–l), while no significant difference was observed for HBA1. It should be noted that analysis of the six proteins identified as a biomarker performed directly on serum did not separate melanoma patients from healthy individuals (Figure S13).

To refine our analysis, we investigated the most representative subgroup of DEP-4 across all proteomics runs. The ideal subgroup was identified based on three criteria: (a) no misclassification when clustering any data set; (b) adjusted *p*-values ≤ 0.05 across all data sets; (c) the highest average silhouette value among all subgroups meeting a and b. Table S3 lists all of the DEP-4 subgroups that satisfy a and b. Among them, a subgroup of only two proteins—THBS1 and SERPING1—stood out with the highest average silhouette value of 0.65. Sample clustering based on these two proteins in the validation run also shows a high silhouette value of 0.75, indicating strong classification credibility. Similar to the six proteins above, PCA based on THBS1 and SERPING1 did not separate melanoma patients from healthy individuals in the serum comparison (Figure S14a). However, PCA based on the two proteins can distinguish melanoma patients from healthy individuals (Figure S14b) when comparing proteomics results from ProLips.

This finding aligns with previous literature, which highlights the roles of both THBS1 and SERPING1 in melanoma progression and metastasis. THBS1 (thrombospondin-1) is a multifunctional glycoprotein known to regulate tumor microenvironments, angiogenesis,^52^ and immune responses.^53^ The up-regulation of THBS1 in melanoma ProLips may reflect its involvement in promoting tumor growth and metastasis.^54^ On the other hand, SERPING1 (C1 inhibitor) is a key regulator of the complement system, and its down-regulation in melanoma ProLips could be linked to immune evasion mechanisms exploited by tumor cells.^55–57^ Together, the distinct expression patterns of these two proteins support their further evaluation as candidate biomarkers for melanoma detection and their relevance to disease mechanisms.

## Discussion

Our results support the existence of an LDL–protein complex in human biofluids and thus suggest that naturally occurring LDL can associate with proteins to form a corona. Different extraction methods can result in different proteins: DGUC-isolated LDL are depleted of much of the soft corona while PEG-induced precipitation followed by SEC purification keeps the associated proteins. We should note that DGUC does not extract protein-free LDL, because ApoB accounted for only 55.1% of total spectral counts (Table S2). This is consistent with previous literature describing DGUC as a high-resolution technique to isolate nanoparticles coated with their corona.^58,59^ Therefore, DGUC-extracted LDL should be better interpreted as LDL with only its hard corona, while ProLips are LDL associated with both hard corona and soft corona. Compared to synthetic nanoparticles, the weak and dynamic interaction of LDL with its soft corona may be related to LDL’s relatively small size and lipid-based surface.^60^

LP and EV isolation methods are often affected by reciprocal coisolation^61^ as previous studies have shown that HDL and LDL can be associated with EVs or incorporated into their protein corona.^62–64^ In nonspecific entropy-driven PEG-induced precipitation, multiple nanoparticles may potentially be precipitated, including VLDL, LDL, EVs, and exosomes. HDL—whose fraction was not collected during SEC purification—appeared to contribute only weakly to the SEC signal, likely because of its smaller size and lower precipitation efficiency under the present conditions, though ApoA1 is not completely absent. The DLS and TEM results suggest that LDL represents the dominant recovered nanoparticles, though minor contamination of VLDL, EVs, or exosomes cannot be fully excluded (Figure 2c,d). However, we must admit that some LDL nanoparticles in ProLips may have been associated with EVs in the circulation. The interaction between LDL and EVs is dynamic and weak, and we are not aware of an established method that can distinguish EV-associated LDL from non-EV-associated LDL.

We also explored the role of PEG-induced precipitation in the extraction by comparing the protein composition of ProLips and that of the corresponding SEC-FPLC fraction collected directly from serum (S1 in Figure S3). The two profiles were highly correlated (Pearson’s *r* > 0.98; Figure S3b), indicating that PEG precipitation does not substantially alter the protein composition of ProLips, even though it may possibly affect the adsorption equilibria of proteins on nanoparticles in serum. This suggests that PEG does not “push” the proteins onto LDL to form the corona, but instead precipitates LDL via depletion forces. Therefore, corona formation is an intrinsic nanoscale property of LDL. The precipitation acts as a first-step purification method that reduces abundant proteins like albumin that are not associated with nanoparticles which reduces the burden on the column during SEC purification.

ProLips showed improved performance when compared with serum in distinguishing between melanoma patients and healthy controls, which may be partly attributed to the physical reduction of abundant proteins. The reduction alleviates abundance suppression^65^ and facilitates the detection of lower-abundance proteins. As shown in Figure S3c,g, the spectral contribution of albumin decreased from 29.9% in serum to 2.7% in ProLips, whereas that of ApoB increased from 1.7% to 4.8%, resulting in an approximately 31-fold reduction in the albumin-to-ApoB spectral ratio. A similar trend was observed for other abundant serum proteins: the relative spectral contributions for transferrin and IgG compared to ApoB decreased by approximately 26-fold and 11-fold, respectively. Consistently, computational removal of signals from these proteins in the serum comparison of healthy controls and melanoma patients did not reproduce the separation observed with ProLips comparison (Figure S6), as post hoc removal cannot recover signals from low-abundance proteins that were not detected during MS acquisition. In addition, enrichment of LDL-associated proteins may further contribute to the improved performance of ProLips. Among all 32 proteins detected in ProLips but not in serum by the label-free proteomics, 20 proteins were significantly up- or down-regulated in ProLips comparison in the initial TMT-based melanoma-versus-healthy study (Figure S7). Among the 110 proteins that account for a higher spectral percentage in ProLips than serum, 80 proteins were significantly up- or down-regulated in TMT-based study.

The cancer-related results should be interpreted as proof-of-concept disease separation and biomarker discovery as they were not obtained from a conventional clinical diagnostic workflow. Some melanoma-vs-healthy comparisons used pooled healthy controls, which may reduce biological variance in the control cohort and contribute to apparent group separation. Larger studies using fully individual, age- and gender-matched cohorts will be required to validate the robustness of the observed signatures. Nevertheless, as the serum and ProLips comparisons in the initial study were performed using samples from the same donors, the design supports the conclusion that ProLips revealed stronger melanoma-associated proteomic differences than whole serum in this cohort. Furthermore, individual-based comparisons in colon carcinoma, liver cancer, and melanoma support the broader conclusion that ProLips proteomes contain much disease-related information.

Large DEP pools observed across different cancer types are valuable as disease-associated ProLips signatures for exploratory use, and may also guide annotation analyses. However, using large DEP pools directly as disease biomarkers would be less practical for clinical translation, because it would require proteomics-based measurement of many proteins, which increases cost and assay complexity. Fewer but more robust proteins, such as the six-protein potential biomarker set or the refined THBS1/SERPING1 candidate pair, are more compatible with conventional assays such as ELISA.

## Conclusions

In conclusion, we have shown that the interaction between LDL and proteins leads to the formation of a protein corona and therefore that a protein corona exists on naturally occurring nanoparticles. We call the complex of LDL and their corona ProLips and show that ProLips contain a diverse set of associated proteins. In stark contrast to serum, most of the abundant proteins do not dominate the proteomic composition. This property is useful because it enables proof-of-concept ProLips-based disease detection and facilitates biomarker discovery. Our findings demonstrate the superiority of ProLips in revealing protein expression differences that are often masked in serum analysis. In fact, by analyzing ProLips extracted from the serum of patients we can successfully reveal differences in proteomic profiles in melanoma, colon carcinoma, or liver cancer patients and healthy people. A statistical analysis of our data led to the identification of a set of six proteins that, taken together, could be used as a novel melanoma biomarker set. Given the relatively small cohort size of 50 samples, we call for more ProLips-related studies in larger, independent cohorts to validate the proposed biomarker set for melanoma and assess its clinical specificity and generalizability.

## Supplementary Material



## Notes

Methods for synthesis, characterization and proteomic analysis, and supplementary experimental results and notes (PDF)
