# Supplementary material for: Characterization and Applications of the Protein Corona Forming on the Naturally Occurring Nanoparticles, Low-Density Lipoproteins
Source: ACS Cent Sci. 2026 Sep 14;12(9):1439–50. doi: 10.1021/acscentsci.6c00872 (PMC13614051; doi:10.1021/acscentsci.6c00872)
Supplement: Supplementary file 1 [file oc6c00872_si_001.pdf]

Supporting Information

Characterization and Applications of the Protein Corona Forming on the Naturally Occurring Nanoparticles, Low-Density Lipoproteins

Ding Ren[1], Lixia Wei[1], Simon Boutry[2], Melis Özkan[1], Ekaterina Poliukhina[1], Laia Simo Riudalbas[2], Krisztian Homicsko[3], Daniel E. Speiser[3], Jacques Fellay[2,4, *], Quy Khac Ong[1, *], Francesco Stellacci[1, 2, 5, *]

[1] Institute of Materials, École Polytechnique Fédérale de Lausanne (EPFL), Lausanne, Switzerland

[2] Global Health Institute, École Polytechnique Fédérale de Lausanne (EPFL), Lausanne, Switzerland

[3] Department of Oncology, University of Lausanne, Lausanne, Switzerland

[4] Biomedical Data Science Center, Lausanne University Hospital and University of Lausanne, Lausanne, Switzerland

[5] Institute of Bioengineering, École Polytechnique Fédérale de Lausanne (EPFL), Lausanne, Switzerland

[*] Email address: jacques.fellay@epfl.ch, quy.ong@epfl.ch, francesco.stellacci@epfl.ch.

Methods

Materials

Polyethylene glycol 10000, sodium dodecyl sulfate (SDS), tris hydrochloride (Tris HCl), urea, tris-(2-carboxyethyl)phosphine (TCEP), 2-chloroacetamide (CAA), calcium chloride ($CaCl_2$) was purchased from Sigma-Aldrich. Phosphate buffer saline (pH = 7.4, without calcium, magnesium, phenol red), NuPAGE™ 4 to 12% Bis-Tris gel, Novex™ Tris-Glycine SDS Sample Buffer (2×), NuPAGE™ LDS Sample Buffer (4×), Invitrogen™ NuPAGE™ Sample Reducing Agent (10×), NuPAGE™ MES SDS Running Buffer (20×), Qubit Protein Assay Kit (100 assays), trypsin (MS Grade), trifluoroacetic acid (TFA), hydroxylamine 50%, TMTpro™ 18 plex, CDS Empore™ SDB-RPS extraction disks were purchased from ThermoFisher Scientific. Chromafil Xtra PES-45/25 syringe filters were purchased from Macherey-Nagel Gmbh & Co. KG. InstantBlue® Coomassie Protein Stain was purchased from Abcam. Formic acid LC-MS grade (FA) was purchased from Merck. Sep-Pak tC18 was purchased from Waters. Microcon-30 kDa was purchased from Millipore. LysC (MS grade) was purchased from Fujifilm. Acetonitrile (LC-MS grade) was purchased from Biosolve.

Healthy human sera (H3667, H4522, H6914, H5667) were purchased from Sigma-Aldrich. They were all from USA males with AB type blood. H3667 was heat-inactivated serum extracted from plasma. H5667 was heat-inactivated serum extracted from clotted whole blood. H4522 was extracted from plasma. H6914 was extracted from clotted whole blood. Patient's sera were obtained from late-stage melanoma patients. The patients were included in the observational study MEMC 188/12 of the

University Hospital Lausanne (CHUV). Upon arrival, the sera were all kept under -20 °C until use. The study protocols were approved by the "Swiss Ethics Committee on research involving humans" of the Canton of Vaud, Switzerland. All patients provided written informed consent.

PEG-Induced Precipitation of protein-rich lipoprotein nanoparticles (ProLips)

Typically, 1 mL of serum (or plasma) was diluted with phosphate-buffered saline (PBS) to a final volume of 10 mL, then centrifuged at 17,000 × g at 4°C for 30 minutes. The supernatant was collected after filtration through a PES filter with a pore size of 0.45 µm. 2 mL of polyethylene glycol solution (50% m/v) (PEG-50%) was added to the supernatant and vortexed to mix thoroughly. The mixture was incubated at 4°C for 1.5 hours without disturbance, followed by centrifugation at 1,800 × g at 4°C for 15 minutes. The sediment was collected and dissolved in 5 mL of PBS to obtain a transparent solution. 1 mL of PEG-50% was added to this solution, vortexed, and incubated at 4°C for 45 minutes. The mixture was centrifuged again at 1,800 × g at 4°C for 15 minutes. The sediment was collected and re-dissolved in 1 mL of PBS, labeled as X.

FPLC-Purification of ProLips.

ProLips was purified from X using size-exclusion chromatography. The fast-protein liquid chromatography (FPLC) workstation consists of an automatic ÄKTA Go FPLC Cytiva, and a HiPrep™ 16/60 Sephacryl™ S-500 HR column. PBS was used as the elution and equilibration buffer. X was injected into the column using a 1 mL capillary

loop, and the flow rate was kept at 0.5 mL min^{-1}. The sample absorption at 280 nm was automatically recorded and applied to group different fractions based on absorption peaks. For collection, the fraction size was kept at 1 mL. Liquids from the same peak were collected and concentrated using Amicon Ultra-15 Centrifugal Filters Ultracel-100K under 1800 × g, 4°C until the total volume was less than 1 mL.

Characterization of ProLips

The protein concentration of the samples was determined by Qubit Protein Assay Kit, 100 assays. The kit was made up of three Qubit Protein Standards, Qubit™ Protein Buffer and Qubit™ Protein Reagent. 10 µL protein reagent was diluted with 1990 µL protein buffer to form precursor A. To measure protein concentration, 2 µL sample and 198 µL A was mixed together to form a transparent solution which was then incubated for 15 minutes under 4ºC. The solution was then tested by Qubit 4 Fluorometer (Thermofisher Scientific, USA).

The morphology of the samples was observed by negative stain electron microscopy (NS-EM). Samples were diluted with PBS 1x to achieve an optimal protein concentration of 0.005-0.01 mg/mL. A sample was adsorbed to a glow-discharged carbon-coated copper grid 200 mesh (CF200-CU-50, EMS, Hatfield, PA, USA) for 2 min followed by washing with deionized water for 30 s and then blotting with a filter paper. Next, the grid was placed on a drop of a solution of uranyl acetate 0.5% for 1 min, and the excess solution was removed by filter paper. The grid was left to dry at room temperature under aluminum foil to avoid exposure to light. The observation was

made using a Talos L120C G2 electron microscope (Thermo Fisher, Hillsboro, USA) operated at 120 kV. Digital images were collected using a Ceta S 16M CMOS camera (Thermo Fisher, Hillsboro, USA) 4096x4096 pixels.

Analytical ultracentrifugation in sedimentation velocity mode (AUC-SV) measurements were performed in an analytical ultracentrifuge XL-I machine x(Beckman-Couter). Samples (370 μL) at the absorbance 0.2 < Optical Density (OD) < 1 was loaded in the sample sector of the 12 mm cell, while PBS 1X (380 μL) was used in the reference sector. The cells were placed in an An60-Ti rotor, equilibrated at 20°C in a vacuum for 2 hours, followed by velocity scans at 18,000 rpm with absorbance profile collected at 280 nm. Data analysis was done in SEDFIT software[1] using the following settings: maximum entropy regularization and a confidence level of 0.68 with a final s-resolution of 600 in s-interval from 0–100 S. A partial specific volume used was 0.73 mL/g, and density and viscosity were measured for the sample, assuming a negligibly small effect of protein due to its low concentration.

Dynamic light scattering (DLS) curves were obtained by a Malvern Nano ZS. Eppendorf Vis Cuvette with light transmission 300-900 nm was used as the container. Typically, the sample was diluted to lower the protein concentration to ~0.2 mg mL^{-1}. 500 µL of the diluted sample was put into the cuvette and scanned for 15 runs (each run lasted 10 seconds) under room temperature.

Sodium dodecyl sulfate-polyacrylamide gel electrophoresis (SDS-PAGE) images were obtained in the following protocol. The volume of the samples needed to obtain 5 µg was calculated with their concentration. The samples were then diluted with PBS 1X to

compensate its volume to 10 µL. For non-reducing SDS-PAGE images, diluted samples were added with 10 µL Novex™ Tris-Glycine SDS sample, then injected to NuPAGE™ 4 to 12% Bis-Tris gel with NuPAGE™ MES SDS Running Buffer as the buffer. Electrophoresis condition was set to 150 V, 120 mA, 150 W for 45 minutes. After carefully stripping the gel off the plastic plate, it was stained by InstantBlue® Coomassie Protein Stain for one hour then washed with deionized water for several hours. The image of the final gel was obtained with GelDoc Go Gel Imaging System (Bio-Rad, CA, USA).

Sample Processing for Mass Spectrometry

Samples were digested by FASP (Filter Aided Sample Preparation) following the standard procedure[2] including minor modifications. Protein samples containing 20 µg in 2% SDS, 0.1 M Tris-HCl pH 8.0 were deposited on conditioned filter devices (30K) and centrifuged at 10’000 x g for 30 min. until complete dryness. Filters were washed twice using same centrifuge conditions with 200 µL of Urea solution (8M Urea, 0.1 M Tris-HCl pH 8.0). Reduction on top of the filters was performed with 100 µL of 10 mM TCEP in 8 M urea, 0.1 M Tris-HCl pH 8.0 at 37°C for 60 minutes with gentle shaking. Removal of reduction solution was performed by extended centrifugation followed by two extra washing steps. Alkylation on top of the filters was performed with 100 µL of 40 mM chloroacetamide in 8 M urea, 0.1 M Tris-HCl pH 8.0 at 37°C for 45 minutes light protected with gentle shaking. After removal of alkylation solution and two additional washing steps with 200 µL of Urea solution, the filters were further washed

twice with 200 µL of 5 mM Tris-HCl pH 8.0. Samples were then digested overnight on top of filters at 37°C with 100 µL of proteolytic solution at 1/50 w/w enzyme-to-protein ratio using a combination of mass spectrometry grade trypsin and LysC supplemented with 10 mM $CaCl_2$. Peptides were then eluted from filters and two extra elutions were performed with a 50 µL of 4% TFA. Resulting peptides were then desalted on SDB-RPS Empore StageTips using the standard protocol[3] and purified peptides were dried by vacuum centrifugation.

For tandem mass tag (TMT) labeling, dried peptides were reconstituted in 10 µL 100 mM HEPES pH 8 and then, 5 µL of TMT solution (25 µg/µL in pure acetonitrile) was directly added. Labeling was achieved with the TMTpro™ 18plex isobaric Mass Tagging Kit (ThermoFisher Scientific) for 90 minutes at room temperature and reactions were then quenched with hydroxylamine to a final concentration of 0.4% (v/v) for 15 min. A minor fraction of TMT labeled samples were then pooled at 1:1 ratio across all samples and a single LC-MS^2 run was performed to ensure correct peptide mixing across each TMT channel to avoid final extra normalization. Quantities of each TMT-labeled sample were then calculated by the control run and final combined samples were desalted using a 100 mg SEP-PAK C18 cartridge according to provider recommendations and dried by vacuum centrifugation. Pooled samples were then fractionated into 12 fractions by Agilent OFF-Gel 3100 system following the manufacturer's instructions. The resulting fractions were desalted again using SDB-RPS Empore StageTips and dried by vacuum centrifugation.

Liquid Chromatography-Mass Spec/ Mass Spec (LC-MS/MS) for Label Free Data

Each sample was resuspended in 2% acetonitrile. 0.1% formic acid and nano-flow separations were performed on a Vanquish Neo UHPLC system on-line connected with an Exploris 480 Mass Spectrometer. Sample trapping and cleaning was done with a capillary pre-column (Acclaim Pepmap C18, 3 µm – 100 Å, 2 cm × 75 µm ID). Analytical separations were performed at 250 nl/min over a 150 min biphasic gradient on a 50 cm long in-house packed capillary column (75 µm ID; ReproSil-Pur C18-AQ 1.9 µm silica beads; Dr. Maisch). Acquisitions were performed through Top Speed Data-Dependent acquisition mode using 2 s cycle time. First MS scans were acquired at a resolution of 60’000 (at 200 m/z) over a scan range of 350 to1’200 m/z. The most intense parent ions were selected and fragmented by High energy Collision Dissociation (HCD) with a Normalised Collision Energy (NCE) of 30% using an isolation window of 1.4 m/z. Fragmented ion scans were acquired with a resolution 15’000 (at 200m/z) and selected ions were then excluded for the following 20 s.

Label Free data Analysis

Raw data were processed using MaxQuant 1.6.10.43[4] against a protein database consisting of the Uniprot Homo Sapiens Reference proteome database (104’557 protein sequences Release2024_01). Carbamidomethylation was set as fixed modification, whereas oxidation (M) and acetylation (Protein N-term) were considered as variable modifications. A maximum of two missed cleavages were allowed and “Match between runs” option was enabled. A minimum of 2 peptides was required for protein

identification and the false discovery rate (FDR) cutoff was set to 0.01 for both peptides and proteins. Label-free quantification and normalisation was performed by Maxquant using the MaxLFQ algorithm, with the standard settings[5]. The statistical analyses of the label-free data were performed using Perseus version 1.6.15.0 from the MaxQuant tool suite[6]. Reverse proteins, potential contaminants and proteins only identified by sites were filtered out. Additional Data visualisation and basic Spectral Count pseudo quantifications were carried out using Scaffold (v. 5.3.3).

Liquid Chromatography-Mass Spec/Mass Spec (LC-MS/MS) for TMT

Each individual fraction was re-suspended in 2% acetonitrile. 0.1% Formic acid and nano-flow separations were performed on a Vanquish Neo UHPLC system online connected with an Orbitrap Ascend Tribrid Mass Spectrometer. Sample trapping and cleaning was done with a capillary pre-column (Acclaim Pepmap C18, 3 µm – 100 Å, 2 cm × 75 µm ID), followed by an analytical separation at 250 nL min^{-1} over 150 min. biphasic gradients on a 50 cm long in-house packed capillary column (75 µm ID, ReproSil-Pur C18-AQ 1.9 µm silica beads, Dr. Maisch). The data was acquired through a Top Speed Data-Dependent acquisition mode using three seconds cycle time. First MS scans were acquired at a resolution of 120,000 (200 m/z), with the most intense parent ions selected and fragmented by High Energy collision Dissociation (HCD) with a Normalized Collision Energy (NCE) of 36% using an isolation window of 0.7 m/z. Fragmented ions scans were acquired with a resolution of 45,000 (200 m/z) and selected ions were then excluded for the following 45 seconds.

Data Normalization and Processing for TMT

Raw data were processed using SEQUESTand MSFragger in Proteome Discoverer v.2.5 against the Uniprot Human Reference Proteome (Uniprot_Human_104557Sequences_LR2024_01 supplemented with classical MaxQuant contaminants). Enzyme specificity was set to trypsin, with a minimum of two peptides made of at least 6 amino acids were required for protein identification. Up to two missed cleavages were allowed, and a 1% FDR cut-off was applied both at peptide and protein identification levels. For the database search, carbamidomethylation (C), TMTpro tags (K and Peptide N termini) were set as fixed modifications, while oxidation (M), phosphorylation (S, T, Y), acetylation (N termini) and glutamine cyclization to pyro-glutamate (N termini) were considered as a variable. The resulting text files were processed through in-house written R scripts (version 3.6.3). Two steps of normalization were applied. The first step of normalization was the sample loading normalization[7]. Assuming that total protein abundances were equal across the TMT channels, each channel's spectra reporter ion intensities were summed, and each channel was scaled according to this sum so that the sum of reporter ion signals per channel equals to the average of the signals across samples. The second step of normalization was the Trimmed M-Mean normalization step by the package EdgeR[8] (version 3.26.8). Providing that the majority of proteins in the samples are non-differentially abundant, this second step calculated normalization factors according to these presumed unchanged protein abundances. proteins with high or low abundances

and proteins with larger or smaller fold-changes were not considered in the second step normalization. P values were calculated by differential protein expression analysis with Bioconductor package Limma of R (version 3.40.6)[9], which were then adjusted with Benjamini-Hochberg multiple testing correction method[10]. Adjusted P values lower than 0.05 (FDR < 0.05) were considered as significant.

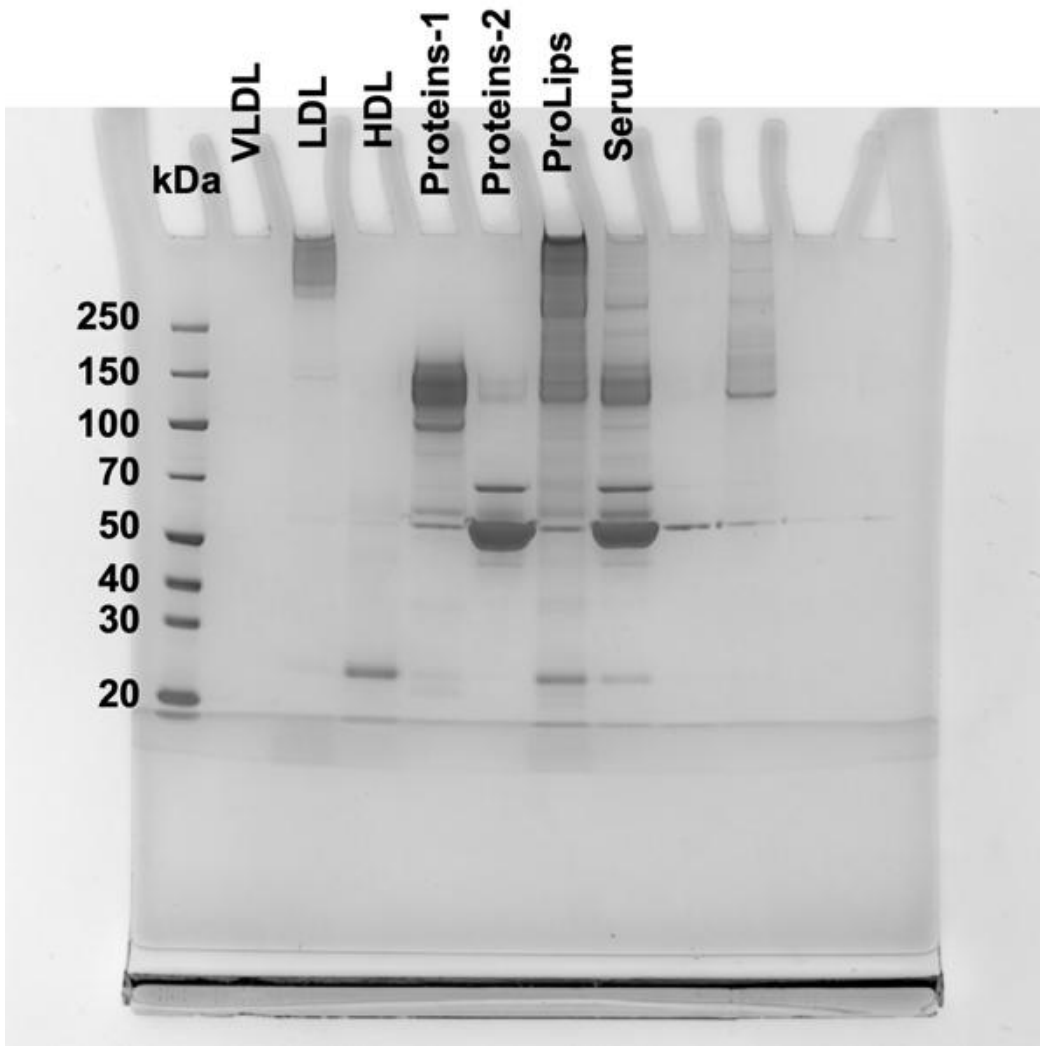

Figure S1. Full SDS-PAGE image of Fig. 1g.

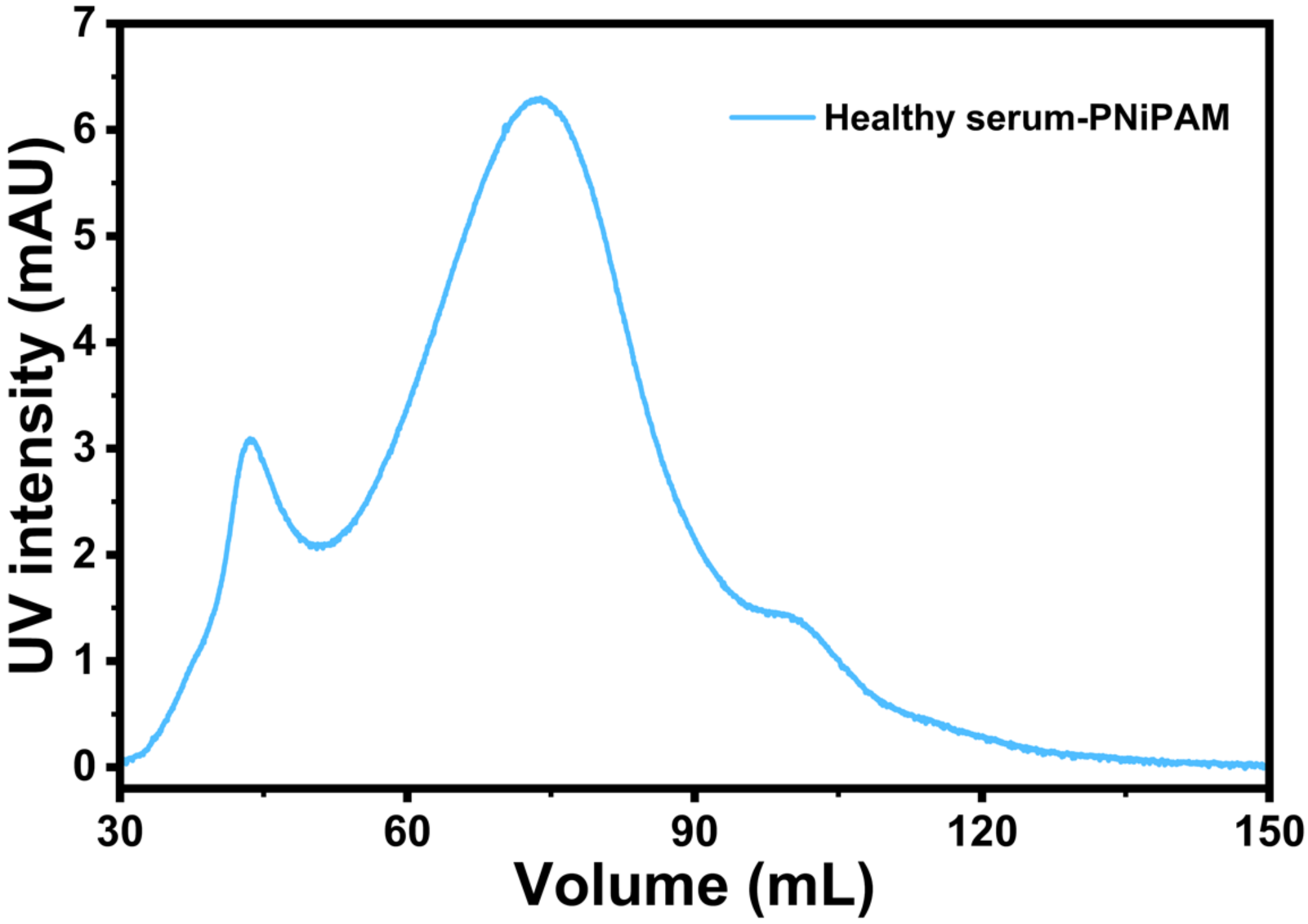

Figure S2. SEC curve of precipitation from healthy serum co-incubated with PNiPAM.

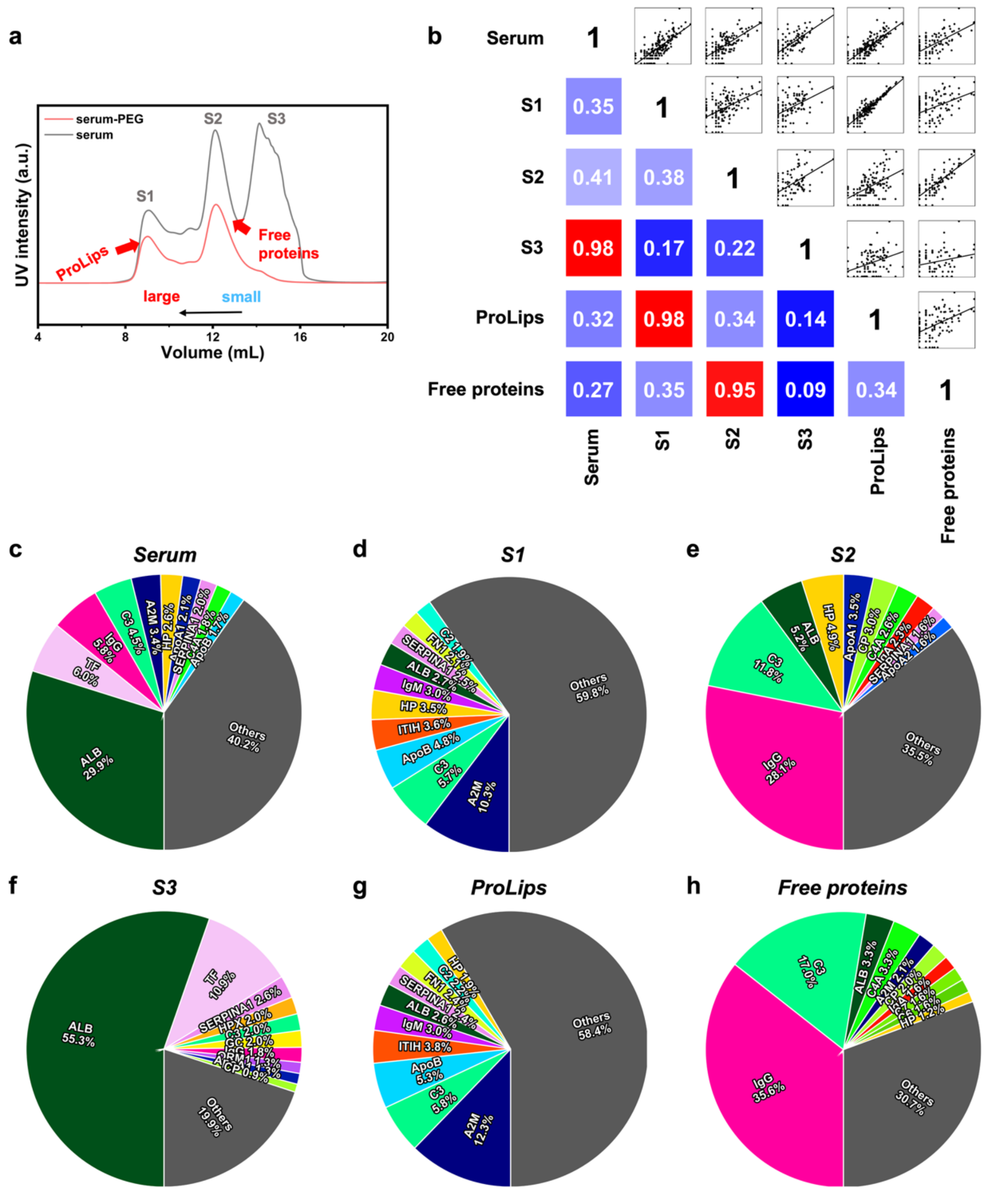

Figure S3. (a) FPLC fractograms of serum (grey) and PEG-precipitated serum (red). Peak fractions collected from serum are labeled S1-S3, and peak fractions from PEG-precipitated serum are annotated as ProLips and Free proteins. (b) Sample-to-sample proteomics correlations shown as a correlation heatmap, with the corresponding scatter

plots and linear regression fits. (c)-(h) Pie charts of the top 10 proteins ranked spectral count percentage of (c) Serum, (d) S1, (e) S2, (f) S3, (g) ProLips, and (h) Free proteins.

Notes: Serum and PEG-precipitated serum were fractionated by FPLC, and peak fractions were collected (Figure S2a). Proteomic profiling was then performed for whole serum, the three serum-derived peak fractions (S1–S3), and the two fractions obtained from PEG-precipitated serum (ProLips and Free proteins). Sample-to-sample correlations (Fig. S2b) show that the protein composition of ProLips correlates well with that of S1, Free proteins is more similar to S2, and whole serum closely matches S3. Spectral-count-based composition summaries further show that albumin and serotransferrin are the two most abundant proteins in whole serum (Fig. S2c) and also dominant in S3 (Fig. S2f) but significantly less prominent in the other fractions (Fig. S2d, e, g, h), indicating that both PEG precipitation and FPLC fractionation is successful in reducing the most abundant serum protein. Furthermore, the close matching of ProLips and S1 prove that PEG precipitation does not significantly change ProLips proteome; instead, it primarily acts as a “first-step filtration” that separates a ProLips-like fraction from abundant proteins, which effectively reduces the ALB burden in the downstream ProLips purification.

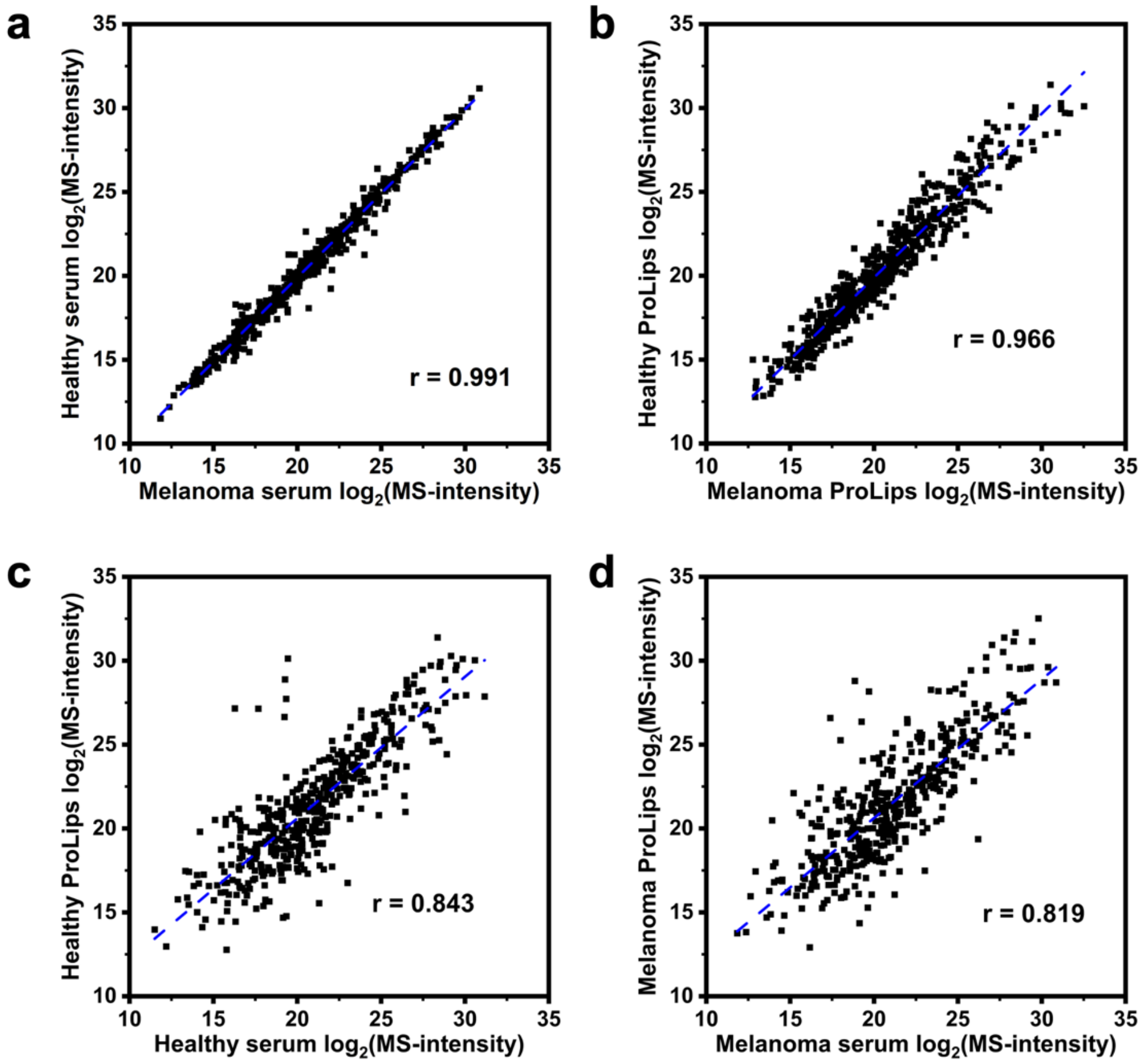

Figure S4. Pairwise-correlations of (a) healthy serum and melanoma serum, (b) healthy ProLips and melanoma ProLips, (c) healthy ProLips and healthy serum, (d) melanoma ProLips and melanoma serum. Blue dotted line were linear fit line and Pearson's r values were calculated.

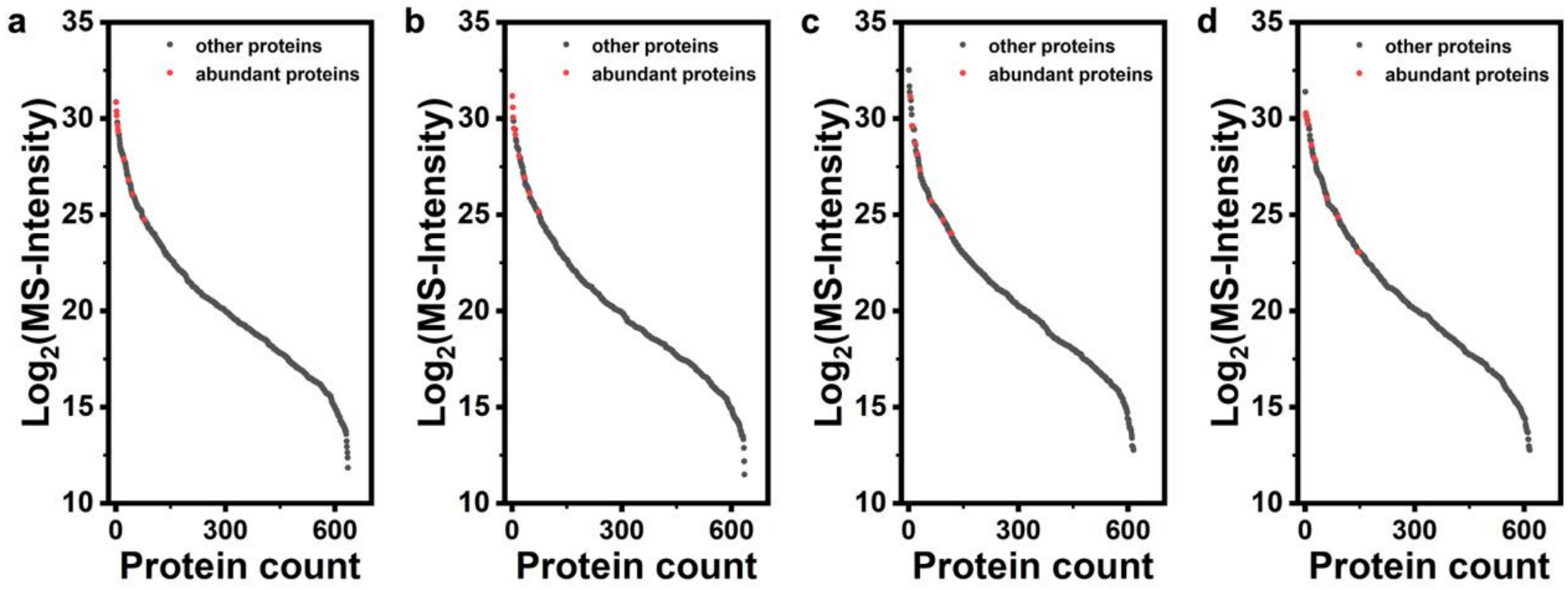

Figure S5. Log_2(intensity) of all proteins from different samples. (a) Melanoma serum, (b) Healthy serum, (c) Melanoma ProLips, (d) Healthy ProLips.

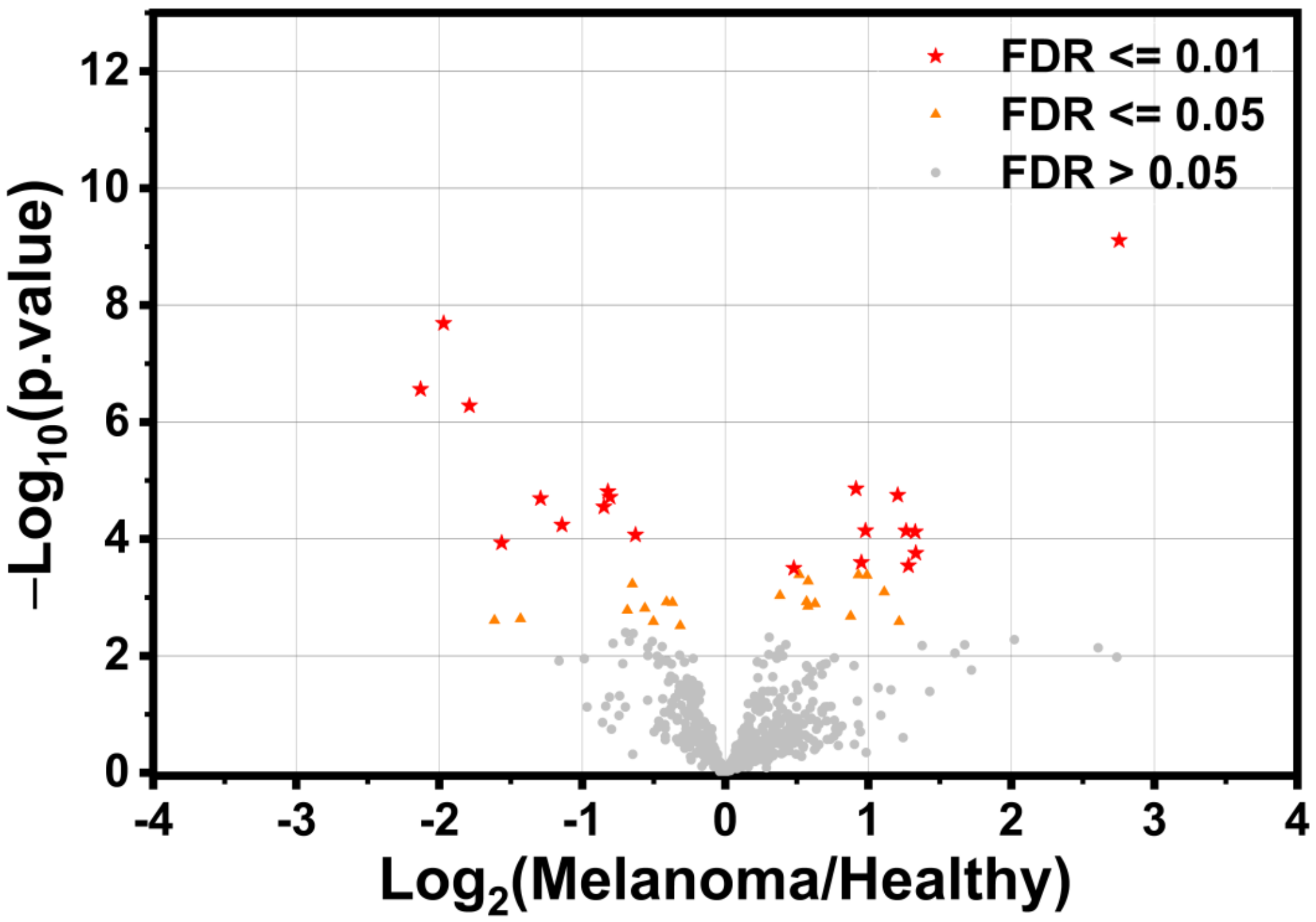

Figure S6. Volcano plot of protein expression comparison on melanoma serum vs healthy serum. Data of albumin, transferrin, and IgG was removed.

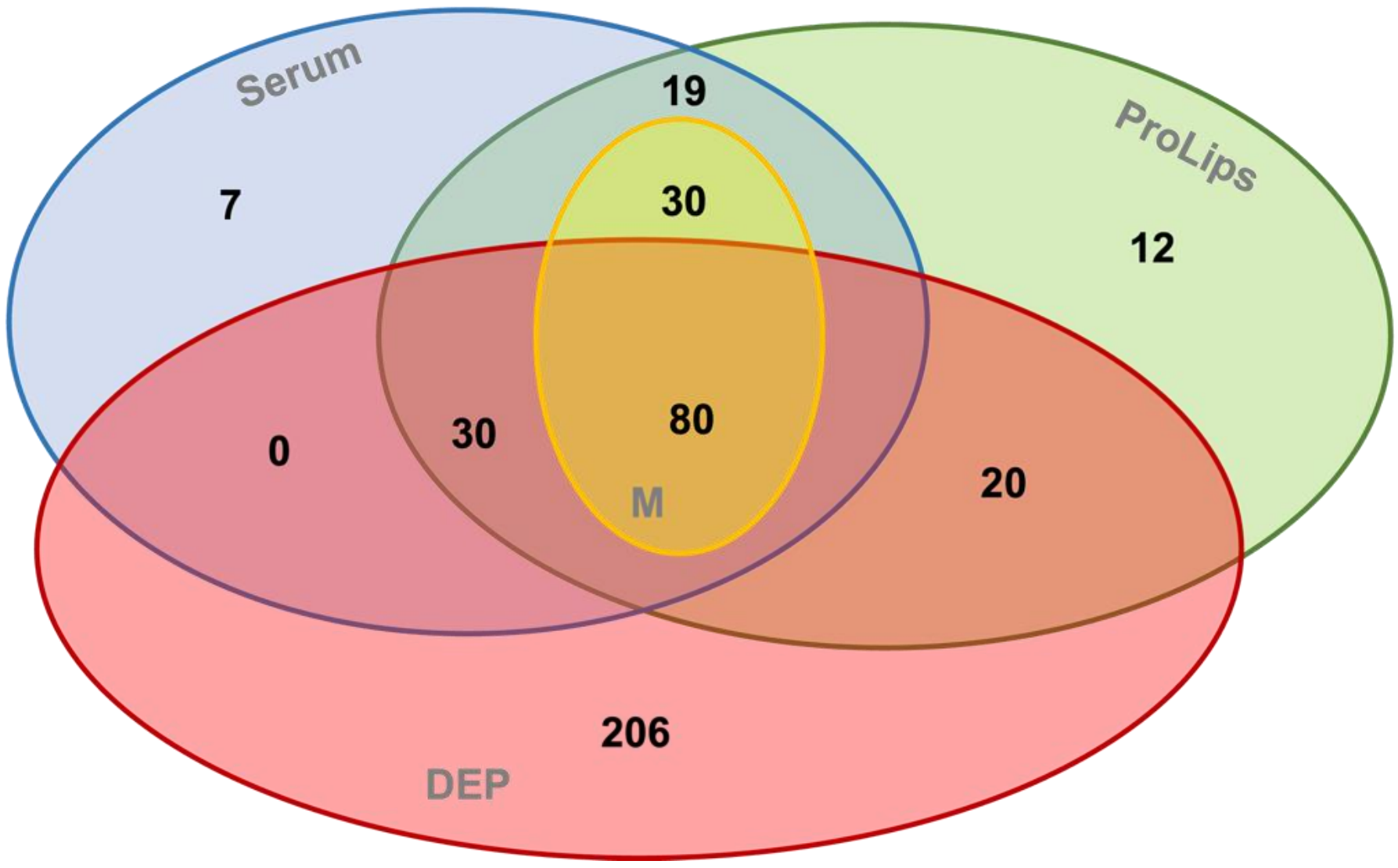

Figure S7. Venn Diagram of detected proteins and DEPs. Here "Serum" represents all proteins detected from serum in label-free proteomics, "ProLips" represents all proteins detected from ProLips in label-free proteomics, "DEP" represents all significantly up- or down-regulated proteins in ProLips comparison in our first melanoma-healthy study, and "M" represents all proteins that were in both "Serum" and "ProLips", but have a higher spectral percentage in ProLips than in Serum.

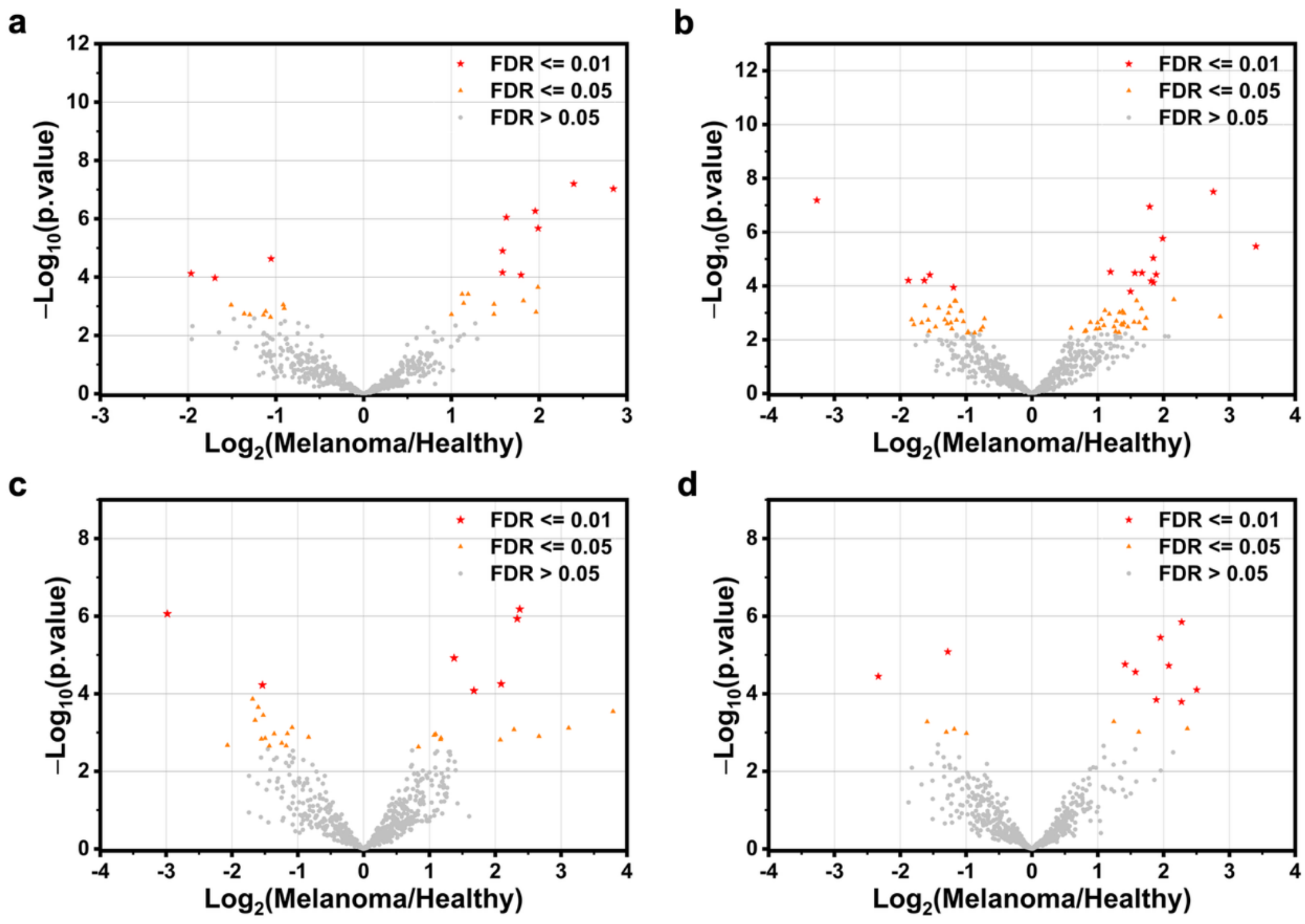

Figure S8. Volcano plots of different pairwise group analysis of melanoma vs healthy.

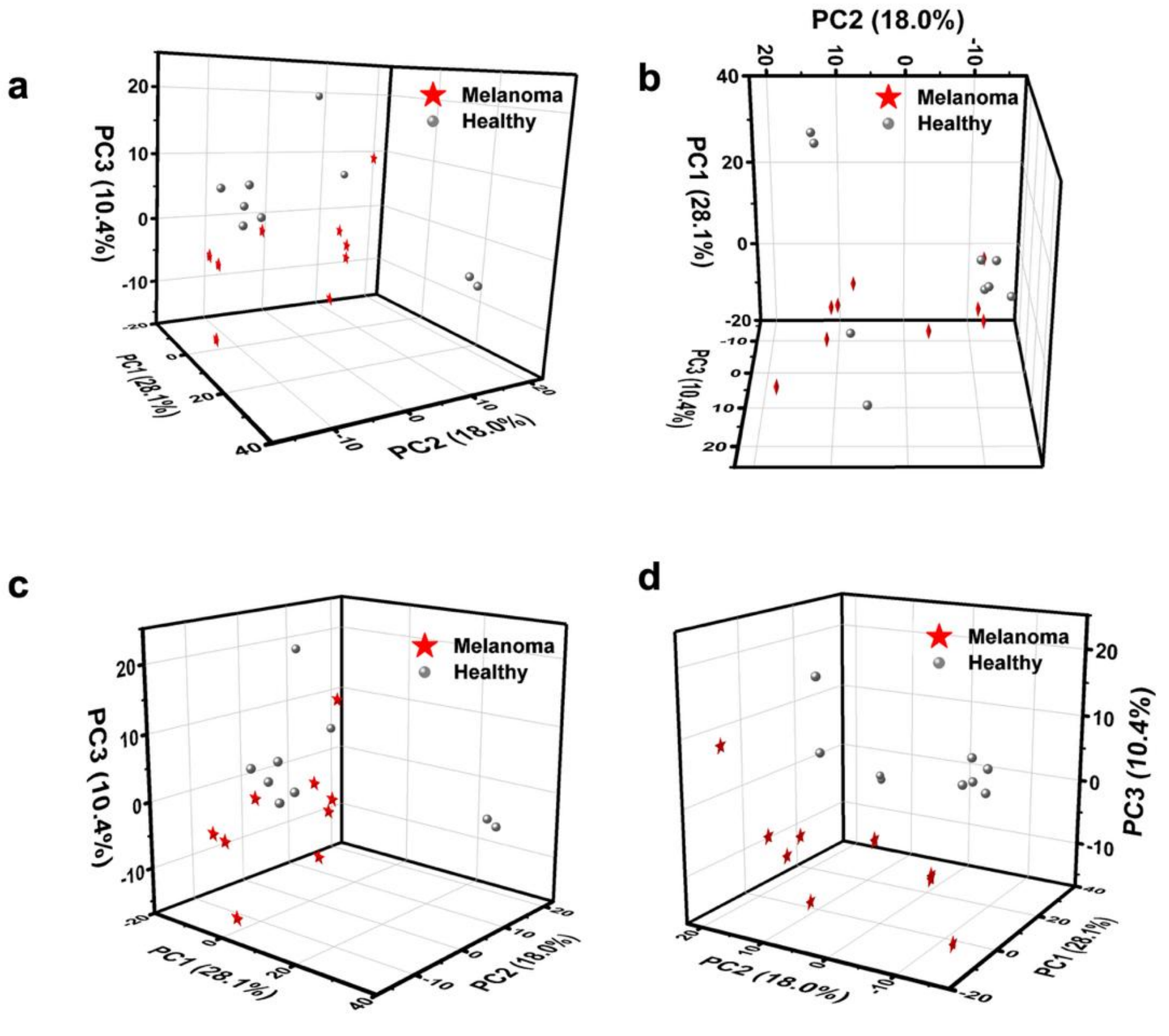

Figure S9. (a)-(d) Different directions of 3D-PCA of ProLips of all samples from the validation run.

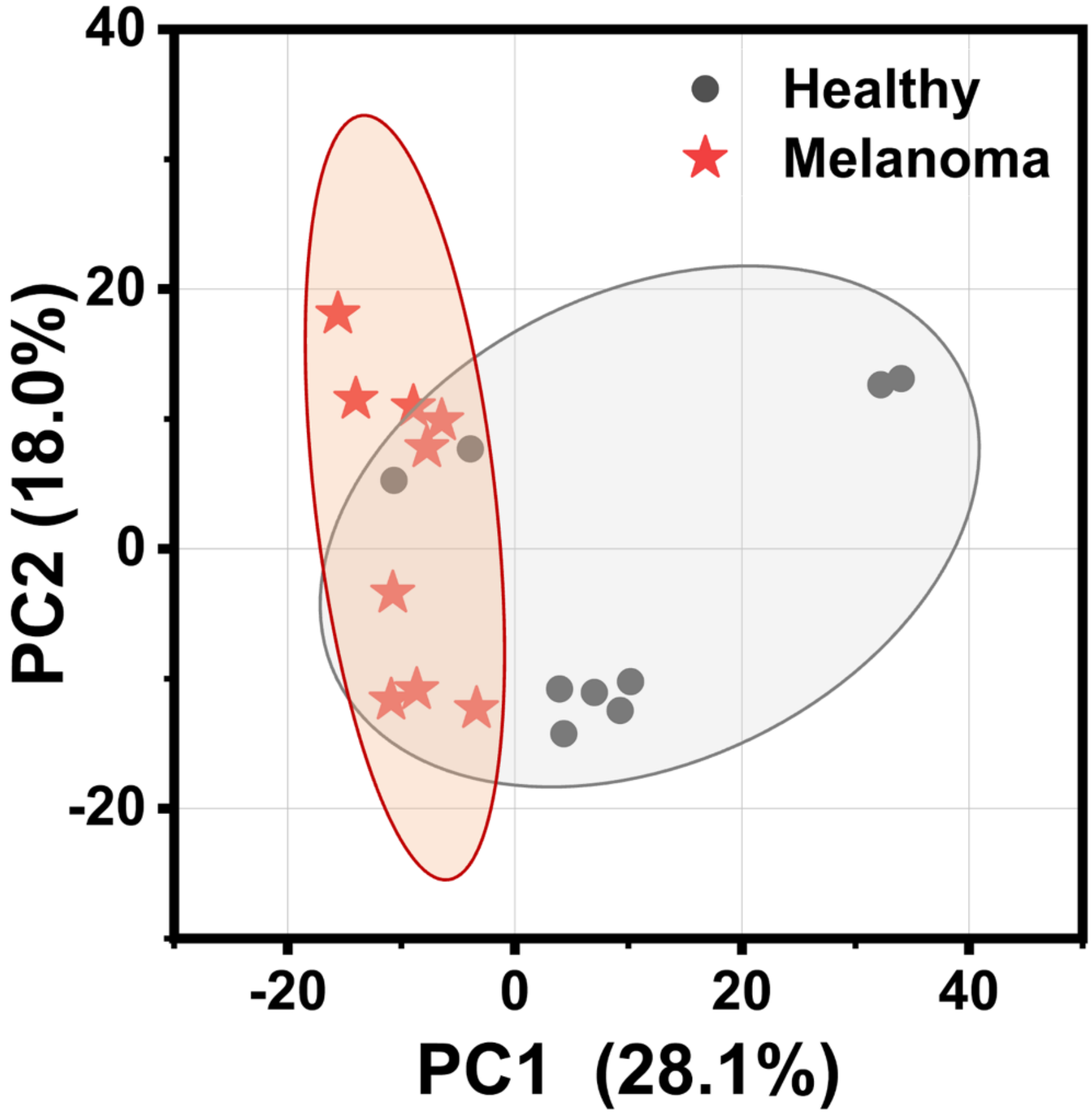

Figure S10. 2D-PCA of ProLips from all samples in the validation run.

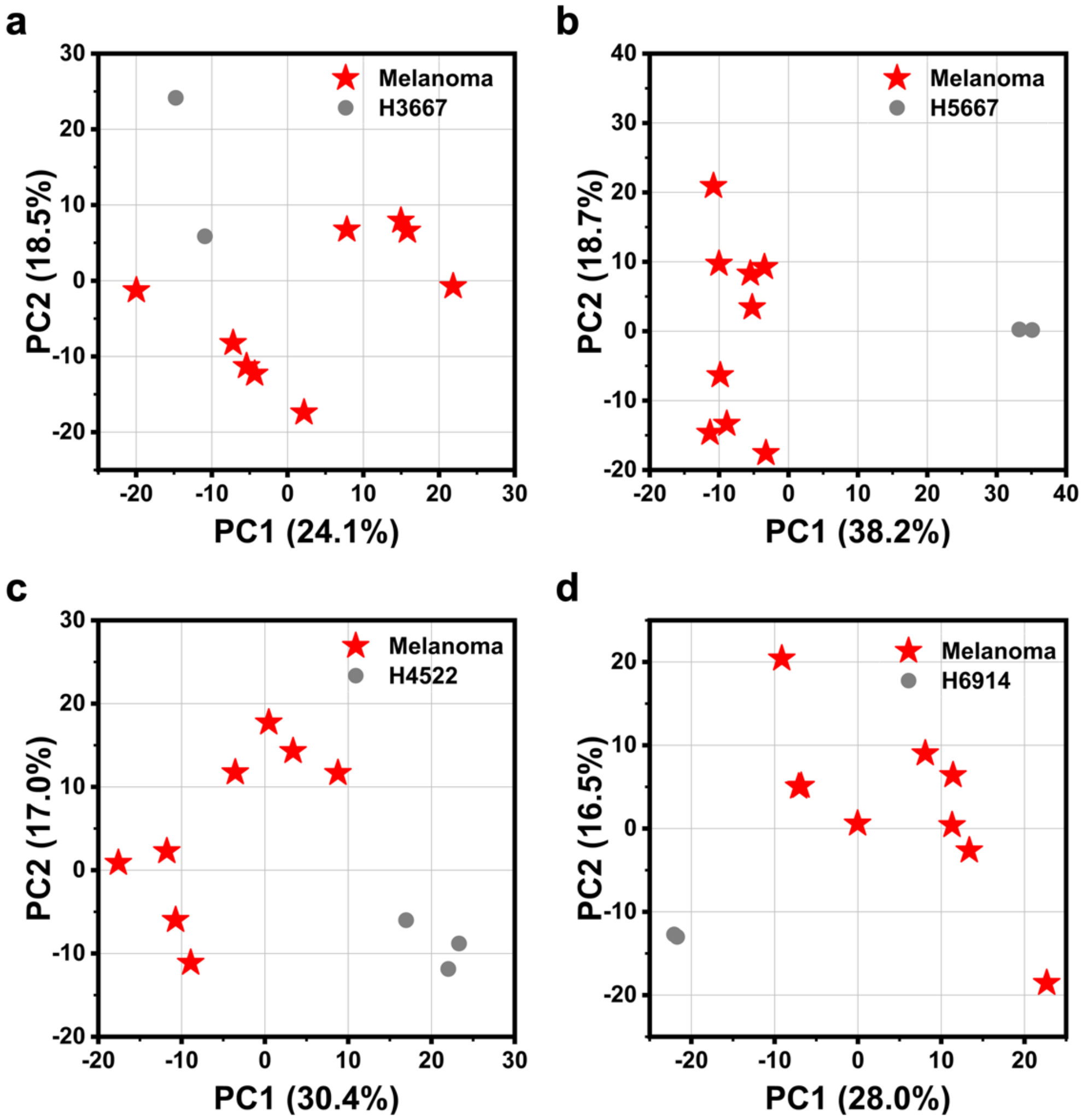

Figure S11. 2D-PCA on protein expression of ProLips from melanoma and each pool.

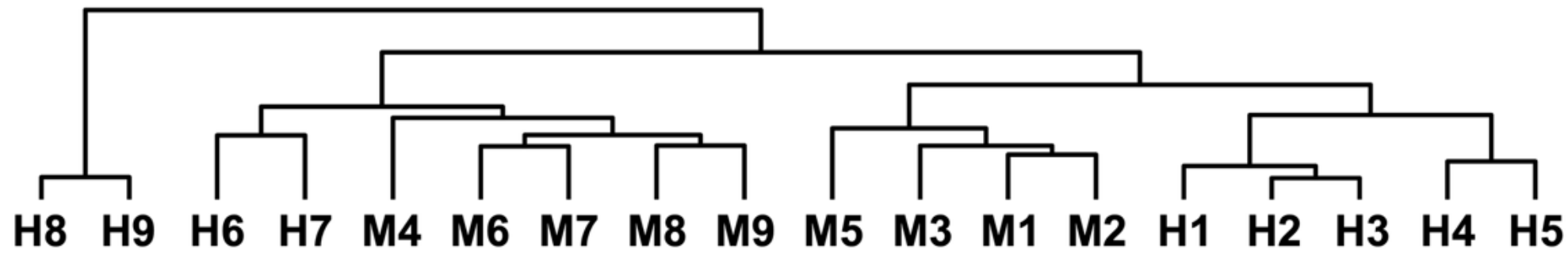

Figure S12. Unsupervised hierarchical clustering of the samples from the validation set based on the expression of all proteins. Here "M" represents ProLips from melanoma samples and "H" represents ProLips from healthy samples.

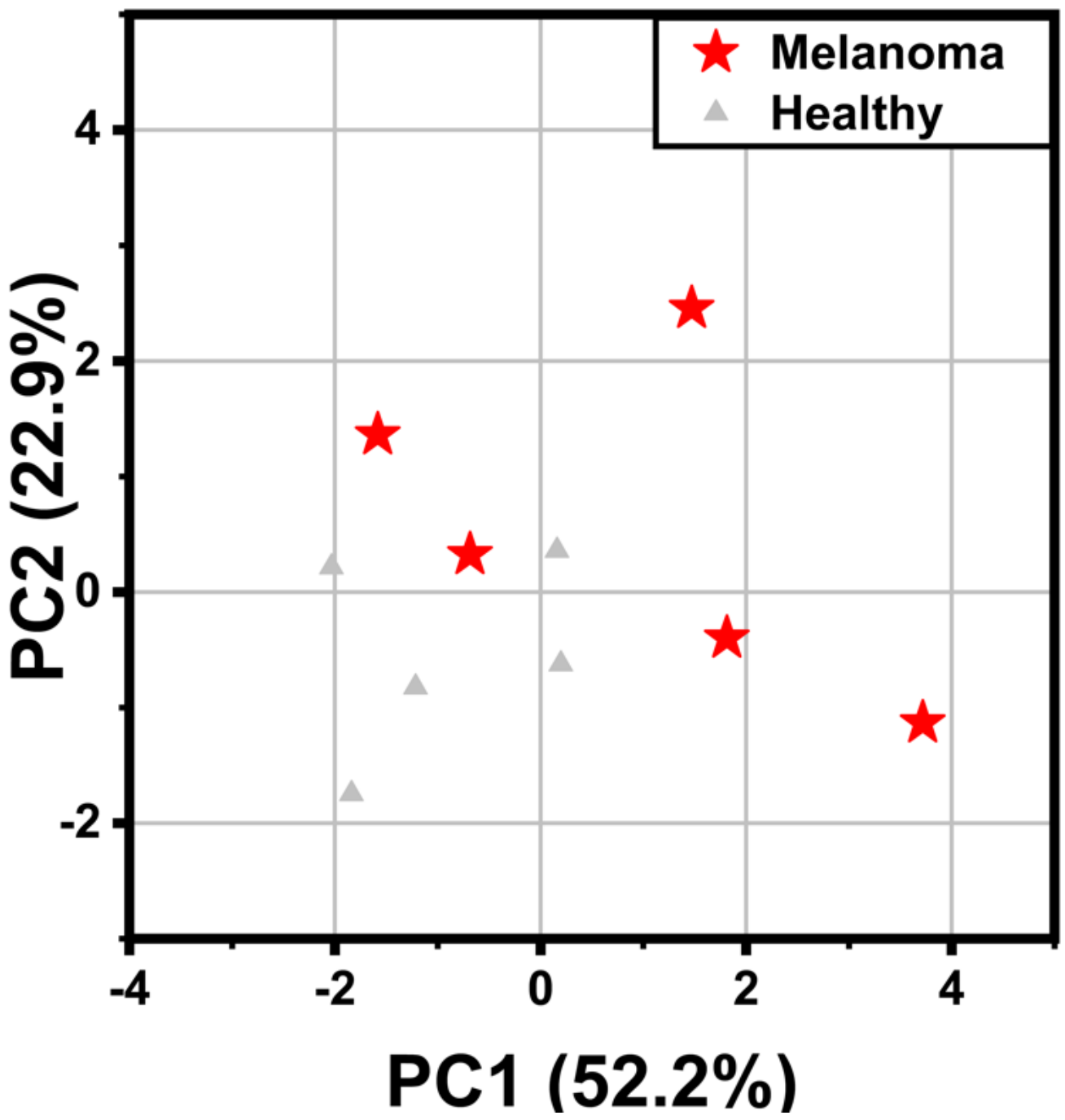

Figure S13. PCA based on the biomarker group (6 proteins) of serum comparison between melanoma individuals and healthy individuals.

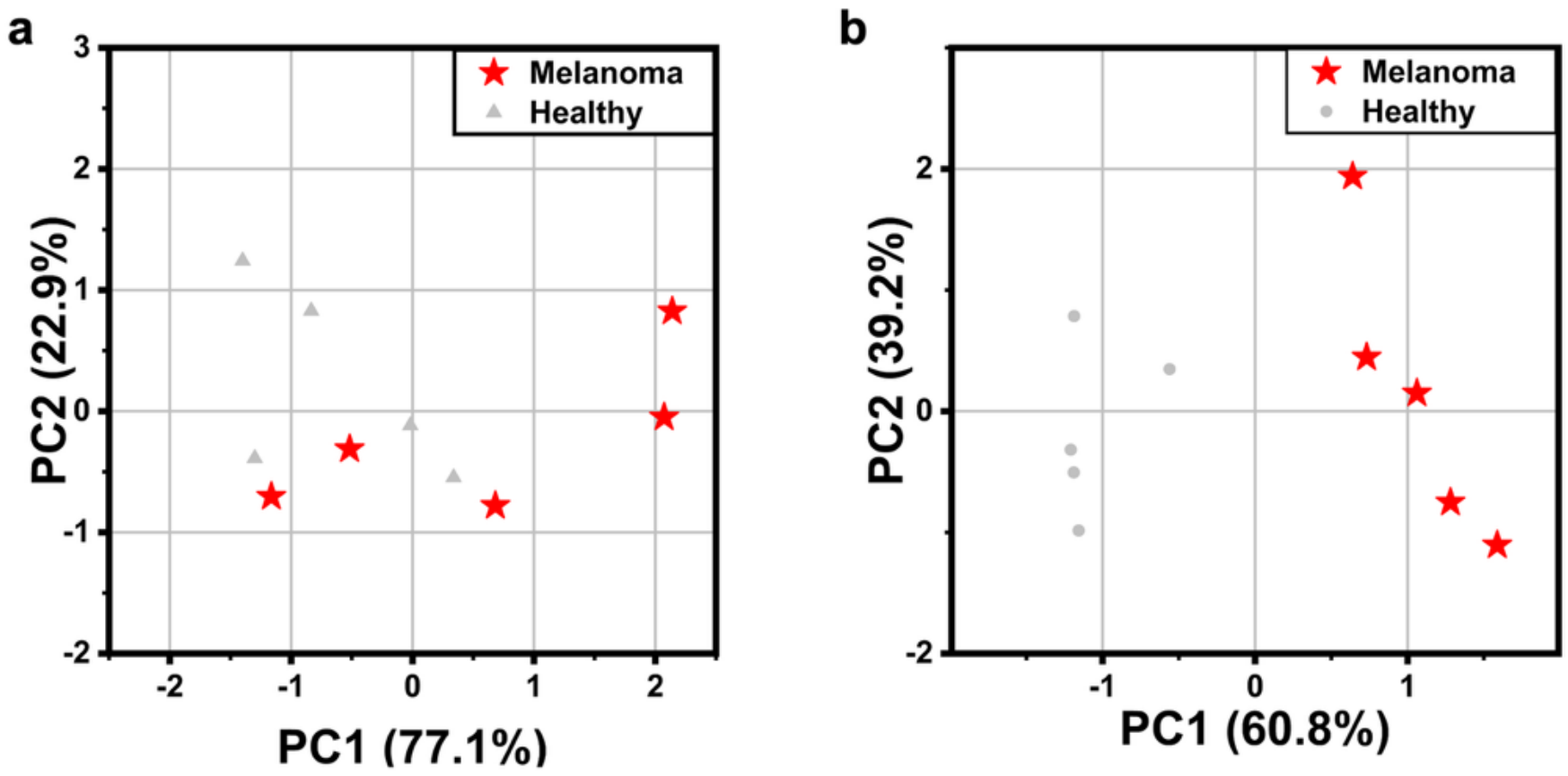

Figure S14. PCA based on THBS1 and SERPING1 of (a) serum comparison and (b) ProLips comparison between melanoma individuals and healthy individuals.

Polymer used	Molecular weight	Concentration (m/v)	Sediments?
PEG	10K	50%	Yes
poly(N-isopropylacrylamide)	10K	40%	Very little
poly(vinyl alcohol)	10K	35%	No
	30K	30%	No
polyacrylamide	40K	25%	No

Table S1. Polymers' ability on precipitating down ProLips.

**Protein**	**Serum**	**ProLips**	**LDL**
Albumin	29.9	2.6	2.42
IgG	6.17	1.64	1.01
Serotransferrin	6.0	0.6	0.78
Complement C3	4.5	5.8	2.44
Alpha-2-macroglobulin	3.4	12.3	1.26
Haptoglobin	2.6	1.9	0.48
Apolipoprotein A-I	2.1	1.3	2.66
Alpha-1-antitrypsin	2.0	2.4	1.47
Apolipoprotein B-100	1.7	5.3	55.10

Table S2. Serum top abundant proteins' spectral percentage in serum, ProLips and LDL in label-free proteomics.

Rank	Proteins	Run 1	Run 2	Run 3	Run 4	Run 5	Run 6	Run 7	Validation Run	Average
1	SERPING1/THBS1	0.62	0.65	0.63	0.66	0.56	0.67	0.64	0.75	0.65
2	CNDP1/SERPING1/THBS1/APP	0.64	0.65	0.60	0.65	0.60	0.65	0.60	0.71	0.64
3	CNDP1/SERPING1/APP	0.67	0.64	0.63	0.65	0.63	0.62	0.57	0.66	0.63
4	SERPING1/APOH/THBS1/APP	0.60	0.65	0.59	0.63	0.62	0.64	0.63	0.69	0.63
5	SERPING1/APOH/APP	0.62	0.64	0.61	0.62	0.65	0.61	0.61	0.63	0.62
6	CNDP1/SERPING1/APOH/THBS1/APP	0.59	0.64	0.55	0.61	0.64	0.62	0.59	0.68	0.61
7	CNDP1/APOH/THBS1/APP	0.56	0.65	0.48	0.61	0.64	0.64	0.60	0.70	0.61
8	CNDP1/SERPING1/APOH/APP	0.61	0.63	0.57	0.61	0.66	0.59	0.56	0.64	0.61
9	SERPING1/APOH/THBS1	0.55	0.62	0.54	0.59	0.65	0.61	0.60	0.69	0.61

Table S3. Silhouette analysis on the classification based on proteins groups refined from all subsets of DEP-4 that met the first two criteria. The protein groups were ranked based on their average silhouette score.

Sample label	Sample type	Individual/Pooled	Health status	Age	Gender	Proteomics runs
7915692251	serum	individual	healthy	58	F	7
7916155682	serum	individual	healthy	72	F	7
7998131815	serum	individual	healthy	68	M	7
7914636662	serum	individual	healthy	71	F	7
7915288014	serum	individual	healthy	66	M	7
MM622	serum	individual	melanoma	66	M	1,6
MM608	serum	individual	melanoma	67	F	1
MM443	serum	individual	melanoma	61	M	1,6
MM854	serum	individual	melanoma	74	F	1
MM556	serum	individual	melanoma	70	M	1,6
MM537	serum	individual	melanoma	53	F	2
MM567	serum	individual	melanoma	55	F	2
MM241	serum	individual	melanoma	52	M	2
MM523	serum	individual	melanoma	58	M	2
MM441	serum	individual	melanoma	66	M	2
MM561	serum	individual	melanoma	69	M	2,4
M309	serum	individual	melanoma	57	F	3
M586	serum	individual	melanoma	50	M	3
M576	serum	individual	melanoma	57	M	3
M648	serum	individual	melanoma	68	M	3

MM552	serum	individual	melanoma	35	F	4
MM314	serum	individual	melanoma	45	F	4
MM562	serum	individual	melanoma	55	M	4
MM359	serum	individual	melanoma	61	M	4
MM446	serum	individual	melanoma	81	M	4
SSM392	serum	individual	melanoma	34	F	5
SSM465	serum	individual	melanoma	49	M	5
SSM321	serum	individual	melanoma	68	M	5
SSM481	serum	individual	melanoma	74	M	5
MM524	serum	individual	melanoma	56	M	6
MM352	serum	individual	melanoma	77	M	6
MM754	serum	individual	melanoma	79	M	6
MM156	serum	individual	melanoma	53	M	6
MM529	serum	individual	melanoma	64	M	6
MM407	serum	individual	melanoma	57	M	6
MM288	serum	individual	melanoma	58	M	6
MM445	serum	individual	melanoma	53	M	6
4660974152	serum	individual	melanoma	67	M	7
4660967982	serum	individual	melanoma	56	M	7
4660853081	serum	individual	melanoma	86	F	7
4660957972	serum	individual	melanoma	84	F	7
4660961833	serum	individual	melanoma	73	F	7

H3667	serum	pooled	healthy	/	/	1,2,3,4,5,6
H5667	serum	pooled	healthy	/	/	2,3,4,5,6
H6914	serum	pooled	healthy	/	/	2,3,4,5,6
H4522	serum	pooled	healthy	/	/	2,3,4,5,6
HS-1	serum	pooled	healthy	/	/	2,3,4,5

Table S4. All the samples involved in melanoma-vs-healthy comparisons in this paper. In the "Proteomics runs" column, the number(s) indicate the number(s) of proteomics analysis a certain sample was involved in. Among those, numbers 1, 6, 7 represent: healthy pooled serum vs melanoma individuals (both serum level and ProLips level), validation run, individual serum comparison (both serum level and ProLips level), respectively. Numbers 2 to 5 represent four ProLips comparison from serum (melanoma vs healthy). These four proteomics runs, together with proteomics run number 1 (ProLips level), are the five independent runs described in "Biomarker Discovery" section.

(1) Schuck, P. Size-distribution analysis of macromolecules by sedimentation velocity ultracentrifugation and Lamm equation modeling. *Biophys. J.* **2000**, *78* (3), 1606-1619, Article. DOI: 10.1016/s0006-3495(00)76713-0.
(2) Wisniewski, J. R.; Zougman, A.; Nagaraj, N.; Mann, M. Universal sample preparation method for proteome analysis. *Nat. Methods* **2009**, *6* (5), 359-U360, Article. DOI: 10.1038/nmeth.1322.
(3) Rappsilber, J.; Mann, M.; Ishihama, Y. Protocol for micro-purification, enrichment, pre-fractionation and storage of peptides for proteomics using StageTips. *Nat. Protoc.* **2007**, *2* (8), 1896-1906, Article. DOI: 10.1038/nprot.2007.261.
(4) Cox, J.; Mann, M. MaxQuant enables high peptide identification rates, individualized p.p.b.-range mass accuracies and proteome-wide protein quantification. *Nature Biotechnology* **2008**, *26* (12), 1367-1372. DOI: 10.1038/nbt.1511.
(5) Cox, J.; Hein, M. Y.; Luber, C. A.; Paron, I.; Nagaraj, N.; Mann, M. Accurate Proteome-wide Label-free Quantification by Delayed Normalization and Maximal Peptide Ratio Extraction, Termed MaxLFQ*. *Mol. Cell. Proteomics* **2014**, *13* (9), 2513-2526. DOI: https://doi.org/10.1074/mcp.M113.031591.
(6) Tyanova, S.; Temu, T.; Sinitcyn, P.; Carlson, A.; Hein, M. Y.; Geiger, T.; Mann, M.; Cox, J. The Perseus computational platform for comprehensive analysis of (prote)omics data. *Nat. Methods* **2016**, *13* (9), 731-740. DOI: 10.1038/nmeth.3901.
(7) Plubell, D. L.; Wilmarth, P. A.; Zhao, Y. Q.; Fenton, A. M.; Minnier, J.; Reddy, A. P.; Klimek, J.; Yang, X.; David, L. L.; Pamir, N. Extended Multiplexing of Tandem Mass Tags (TMT) Labeling Reveals Age and High Fat Diet Specific Proteome Changes in Mouse Epididymal Adipose Tissue. *Mol. Cell. Proteomics* **2017**, *16* (5), 873-890, Article. DOI: 10.1074/mcp.M116.065524.
(8) Robinson, M. D.; McCarthy, D. J.; Smyth, G. K. edgeR: a Bioconductor package for differential expression analysis of digital gene expression data. *Bioinformatics* **2010**, *26* (1), 139-140, Article. DOI: 10.1093/bioinformatics/btp616.
(9) Ritchie, M. E.; Phipson, B.; Wu, D.; Hu, Y. F.; Law, C. W.; Shi, W.; Smyth, G. K. <i>limma</i> powers differential expression analyses for RNA-sequencing and microarray studies. *Nucleic Acids Res.* **2015**, *43* (7), 13, Article. DOI: 10.1093/nar/gkv007.
(10) Klipperaurbach, Y.; Wasserman, M.; Braunspiegelweintrob, N.; Borstein, D.; Peleg, S.; Assa, S.; Karp, M.; Benjamini, Y.; Hochberg, Y.; Laron, Z. Mathematical formulas for the prediction of the residual beta cell function during the first two years of disease in children and adolescents with insulin-dependent diabetes mellitus. *Med. Hypotheses* **1995**, *45* (5), 486-490, Article. DOI: 10.1016/0306-

9877(95)90228-7.
